# Toward cardiac electrophysiology digital twins with an efficient open source scalable solver on GPU clusters

**DOI:** 10.1038/s41598-025-33709-w

**Published:** 2026-02-18

**Authors:** Lucas Arantes Berg, Rafael Sachetto Oliveira, Julia Camps, Lucas Marins Ramalho de Lima, Joventino de Oliveira Campos, Zhinuo Jenny Wang, Ruben Doste, Alfonso Bueno-Orovio, Rodrigo Weber dos Santos, Blanca Rodriguez

**Affiliations:** 1 Department of Computer Science, University of Oxford, Oxford, UK; 2 Department of Computer Science, Universidade Federal de São João del-Rei, São João del-Rei, Brazil; 3 Department of Engineering, Universitat Pompeu Fabra, Barcelona, Spain; 4 Graduate Program in Computational Modelling, Universidade Federal de Juiz de Fora, Juiz de Fora, Brazil

**Keywords:** Cardiac digital twin, Purkinje network, Monodomain model, GPU solver, Finite volume method, Open-source software, Software, Biomedical engineering

## Abstract

Modelling and simulation are essential in biomedicine, and specifically in computational cardiology. Reliable, efficient and accurate solvers are critical. This study presents an open-source, GPU-based cardiac electrophysiology solver for scalable multiscale simulations (monoalg3d), incorporating conduction system calibration and performance optimization. The solver employs the monodomain equation coupled with the Purkinje network, solved via the finite volume method, featuring a GPU-based linear solver and concurrent simulation dispatch with MPI. We demonstrate a $10.94\times$ speedup over a CPU-based solution and scalability by running 512 simulations on 128 compute nodes. Coarse and fine biventricular mesh simulations with 855, 670 and 6, 845, 360 control volumes are completed in less than 24 min and 303 min, respectively, considering a single beat and a human-based ventricular cellular model with 43 state variables. The proposed open-source solver enhances computational efficiency and physiological fidelity through Purkinje-muscle-junction calibration, enabling large-scale, high-speed cardiac simulations including the conduction system. This work marks a significant step toward fast and scalable cardiac simulations on GPU architectures by providing execution of concurrent simulations with the novel MPI batch feature and calibration of Purkinje coupling parameters, paving the way for integration into a Digital Twin personalisation pipeline, including the conduction system.

## Introduction

Computational modelling and simulation techniques in biomedicine have advanced over the last decades, from enabling investigations of disease mechanisms to making in silico trials for therapy evaluation possible^1–3^. Computational cardiology is a field that exemplifies a substantial amount of progress^4–7^. Credible computer models of the heart are now available from subcellular to whole-organ dynamics, and efficient and accurate solvers have also been made available enabling large simulation studies. These two advances have driven the field forward towards the realisation of the ‘Digital Twin’ vision in healthcare^7–10^ and in silico trials for therapy evaluation^11^. For this, cardiac models need to incorporate clinically-relevant features of cardiac function and structure, such as the Purkinje conduction system, fibre orientation, anisotropy, cell coupling, and ECG computation, among others. Moreover, in silico clinical trials and therapy evaluation require consideration of large cohorts of virtual patients. Given ecological and economical limitations in computational resources, simulation software needs to provide an accurate and efficient approximation to the mathematical models describing such phenomena. Finally for reproducibility purposes, the software and models need to be made open-source.

The challenge of the high computational costs associated with the resolution of cardiac models has led to the development of more efficient numerical schemes and the adoption of parallel computing techniques to reduce simulation times. In addition, the inclusion of the Purkinje system is a fundamental step toward physiological accuracy and correct modelling of ventricular activation in cardiac digital twin models. In experimental and computational studies, it has been shown that this structure can initiate and maintain certain types of arrhythmias due to altered conduction properties under pathological conditions leading to ectopic beats and reentrant circuits^12^.

Equally important, open-source software presents several advantages^13^: transparency (as the source code is freely available to the public, allowing end-users to validate and verify its functionalities), as well as flexibility and modularity (users can adapt and customise the software to their particular needs by adding novel features or implementing new modules that can be shared in a collaborative development environment).

A range of cardiac solvers has been developed to simulate the heart’s electrical activity. For example, the open-source software opencarp^14^ provides a flexible and parallelized finite element framework but currently lacks support for the Purkinje conduction system. Meanwhile, recent GPU-based solvers such as cardiomat^15^ offer significant acceleration and include Purkinje modelling, but rely on conditionally stable schemes with strict timestep limits and lack physiological delays at the Purkinje-muscle junctions. While each tool presents trade-offs, GPU cardiac solvers^16–25^ offer improved accessibility and cost-efficiency.

monoalg3d uses the finite volume method (FVM) to simulate the monodomain model on GPU and/or CPU hardware, and OpenMP and NVIDIA CUDA to respectively parallelise CPU/GPU computations. In this study, we aim to extend and enhance the open-source solver monoalg3d with novel features. Our goals include: (1) a fully integrated Purkinje network model with a detailed mathematical formulation and Purkinje-muscle-junction parameter calibration; (2) a GPU-based solver for the diffusion linear system, significantly reducing computational time; and (3) an MPI-based batch feature that enables parallel dispatching of simulations across exascale HPC infrastructures. It is important to highlight that with goal (1) we expand on the initial monoalg3d versions presented in Berg et al.^26^ and Riebel et al.^27^, which mostly relies on the Purkinje network generation method and on retrograde propagation through the Purkinje with its potential role in promoting and sustaining complex arrhythmias, respectively. Relative to the original implementation from Oliveira et al.^28^ the novel features this work presents are: a detailed Purkinje coupling modelling, a GPU-based linear system solver, and an MPI-driven batch simulation system. In addition, we improved the input/output of the simulator by using an optimized output format (Ensight) to reduce disk usage. Together, these improvements facilitate large-scale, high-throughput simulation studies critical for digital twin and personalized medicine applications.

Performance improvements are evaluated on different hybrid CPU/GPU combinations for the device and on two human-based cell models of increasing complexity, as well as compared to space adaptivity features presented in previous work^28^. Finally, to demonstrate the full capabilities of the proposed solver and verify its scalability on GPU clusters under more realistic scenarios, our last experiment presents a patient-specific application considering a biventricular simulation with the Purkinje system and ECG recordings.

## Methods

### Monodomain model

The monodomain model is commonly used to describe electrical propagation due to its lower computational cost compared to the bidomain model^22,29^. In the next equations we present the mathematical models for the myocardium and the Purkinje system, along with their coupling. We use subscripts *P* for the Purkinje domain, *M* for the myocardium domain, and $d \in \{P,M\}$ for the full domain.

1 $$\begin{aligned} \beta \left( C_m \frac{\partial V_d}{\partial t} + I_{{ion}_d}(V_d,\vec {\eta _d}) \right)&= \nabla \cdot (\mathbf {\sigma _d} \nabla V_d) + \beta I_{{stim}_d} \ \qquad in \quad \Omega _{d} \times (0, T), \end{aligned}$$

2 $$\begin{aligned} \frac{\partial \vec {\eta _d}}{\partial t}&= f_d(V_d,\vec {\eta _d}) \ \ \ \qquad \qquad \qquad in \quad \Omega _{d} \times (0, T), \end{aligned}$$

3 $$\begin{aligned} \left( \mathbf {\sigma _M} \nabla V_M \right) \cdot \vec {n_M}&= \left( \mathbf {\sigma _P} \nabla V_P \right) \cdot \vec {n_P} \ \ \quad \qquad \qquad on \quad \partial \Gamma _{PMJ} \times (0,T), \end{aligned}$$

4 $$\begin{aligned} \left( \mathbf {\sigma _d} \nabla V_d \right) \cdot \vec {n_M}&= 0 \ \ \quad \qquad \qquad \quad \quad \quad \quad \ \ \ on \quad \partial \Gamma _{d} \times (0,T), \end{aligned}$$

5 $$\begin{aligned} V_d(X_d, 0) = V_{d,0}(X_d), \,\, \eta _d(X_d, 0)&= \eta _{d,0}(X_d) \ \quad \qquad \qquad \qquad X_d \quad in \quad \Omega _d, \end{aligned}$$

where $V_d$ is the transmembrane potential of either domain, $I_{ion_d}$ the total ionic current associated to the cellular model that depends on state variables $\vec {\eta _d}$, $f_d$ the non-linear system of equations encapsulating the dynamics of the state variables, $\beta$ the surface-to-volume ratio, $C_m$ the membrane capacitance, $\mathbf {\sigma _d}$ the domain conductivity tensor, and $I_{stim_d}$ an external stimulus. The model is further closed with appropriate Neumann boundary conditions to ensure flux continuity between the myocardium and Purkinje domains as given by Eq. (3), where $\vec {n_d}$ is the normal vector of the myocardium or Purkinje on the surface interface between myocardium and Purkinje, $\Gamma _{PMJ}$. For the other boundaries, $\Gamma _d$, we simply use standard no-flux boundary conditions. Initial conditions are provided by equation (5). For the Purkinje domain, we consider the one-dimensional form of Eq. (1), while for the myocardium domain, its three-dimensional formulation.

Cardiac tissue is known to be comprised of strongly coupled fibres with anisotropic conduction properties. Such fibres are defined for each myocardial element by three orthonormal vectors ($\vec {f}, \vec {s}, \vec {n}$), where $\vec {f}$ lies on the local fibre or longitudinal direction, $\vec {s}$ on the sheet or transversal direction, and $\vec {n}$ on the normal direction to the fibre. Moreover, associated with each of these vectors, there exist conductivity values $\sigma _{f}$, $\sigma _{t}$, and $\sigma _{n}$, jointly defining the myocardial conductivity tensor as:

6 $$\begin{aligned} \sigma _M = (\vec {f} \otimes \vec {f}) \sigma _{f} + (\vec {s} \otimes \vec {s}) \sigma _{t} + (\vec {n} \otimes \vec {n}) \sigma _{n}. \end{aligned}$$

### Finite volume method applied to the monodomain model

A common technique to efficiently solve the monodomain model is to divide its reaction and diffusion parts using the Godunov operator splitting^30^. Applied to Eqs. (1)–(2), this leads to the solution of two separate problems: a non-linear system of ordinary differential equations (ODEs)

7 $$\begin{aligned} \frac{\partial V_d}{\partial t}&= \frac{1}{C_{m}} \left[ -I_{{ion}_d}(V_d,\vec {\eta _d}) + I_{{stim}_d} \right] , \end{aligned}$$

8 $$\begin{aligned} \frac{\partial \vec {\eta _d}}{\partial t}&= f_d (V_d,\vec {\eta _d}), \end{aligned}$$

and a parabolic linear partial differential equation (PDE)

9 $$\begin{aligned} \beta C_m \frac{\partial V_d}{\partial t} = \nabla \cdot (\mathbf {\sigma _d} \nabla V_d). \end{aligned}$$

Within the different numerical techniques available, the FVM offers a robust approach for solving the monodomain model due to its foundation on conservative principles and applicability to diverse geometries^31^. This technique discretises the computational domain into control volumes. Each control volume is associated with a variable of interest, and the governing equations are applied to ensure the conservation of this variable across the control volume faces.

#### Cell model

For the solution of the cellular electrophysiology model described by Eqs. (7,8) monoalg3d offers different techniques for integration. It supports both the explicit Euler method as well as Rush-Larsen or other methods based on the generalization of matrix exponential, such as the Uniformization approach^32^.

#### Myocardium modelling

To spatially discretise the diffusion term in Eq. (9), we consider the relation:

10 $$\begin{aligned} J_{d} = -\sigma _{d} \nabla V_{d}, \end{aligned}$$

where $J_{d}~(\mu A/cm^{2})$ represents the density of intracellular current flow.

Integrating over each control volume $V_i$, applying the divergence theorem and using equation (9), it yields:

11 $$\begin{aligned} \beta C_m \int _{V_{i}} \frac{\partial V_{d}}{\partial t} dV = - \int _{S_{i}} J_{d} \cdot \vec {n_{d}} \, dA, \end{aligned}$$

where $\vec {n_{d}}$ represents the normal vector to the domain surface. This equation is the basic term for deriving the linear system of equations associated with the linear PDE.

We now particularise the FVM equations for the myocardium. For simplicity, let us consider a tridimensional uniform mesh, consisting of hexahedra with a fixed space discretisation $h_M$. Located at the centre of each myocardial volume (*i*, *j*, *k*) is a node with the transmembrane potential $V_M$ as the associated variable of interest. Assuming that the volumetric membrane current represents an averaged value in each hexahedron, and using (11), we then have:

12 $$\begin{aligned} \left( \beta C_{m} \frac{\partial V_M}{\partial t} \right) \Bigg |_{(i,j,k)} = \frac{- \int _{S_{i}} J_M \cdot \vec {n_M} \,dA}{h_{M}^{3}}. \end{aligned}$$

To support spatially varying fibre orientation and anisotropy of the myocardial conductivity tensor, the surface integral calculations in Eq. (12) consider the total sum of flows on the 6 faces of the control volume (each with face area $h_{M}^{2}$) over a 27-neighbours stencil. This gives:

13 $$\begin{aligned} h_{M}^{3} \beta C_{m} \frac{\partial V_M}{\partial t} = h_{M}^{2} \sum _{l=1}^{6} J_{l}. \end{aligned}$$

Each $J_{l}$ in Eq. (13) is implicitly calculated by evaluating the spatial derivatives of $V_M$ via second-order finite differences at timestep $n+1$, and computing the average conductivity tensor given by (6) at the surfaces of the discretised volume. Altogether, the previous steps lead to a linear system to solve the diffusion equation using the backward Euler method. Additional details are provided in Supplementary Material section A.1 (see Supplementary Figs. S1 and S2).

#### Purkinje modelling

To model the Purkinje system, we consider the one-dimensional form of the linear PDE given by Eq. (9), with time and space discretisations following an equivalent approach to the myocardial case presented above.

**Fig. 1:**
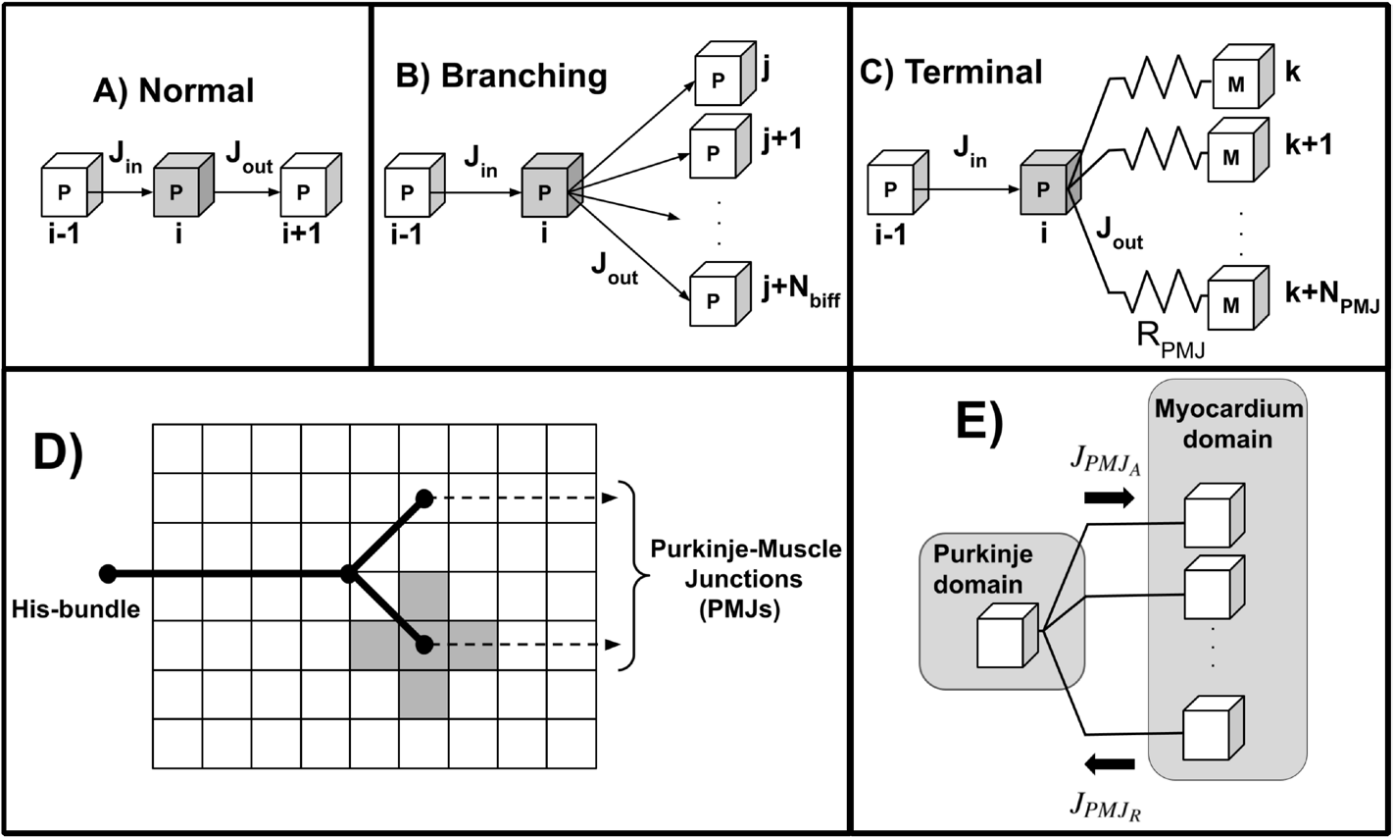
Illustration of the three possible configurations for a Purkinje control volume and the Purkinje coupling model. In panels (**A–C**), the control volume for which we are calculating the fluxes is depicted in grey. (**A**) Normal case, where a Purkinje control volume is associated with no branch. (**B**) Branching case, where a Purkinje control volume is linked to $N_{biff}$ other Purkinje control volumes. (**C**) Terminal case, where a Purkinje control volume is coupled to $N_{PMJ}$ myocardium control volumes from the myocardium domain by a fixed resistance $R_{PMJ}$. Labels *P* and *M* indicate the domain where each control volume is located. (**D**) Simple Purkinje network with single bifurcation, coupling a Purkinje terminal to its five closest myocardium control volumes (coloured in grey). (**E**) Direction of the flux $J_{PMJ}$ for anterograde ($J_{{PMJ}_{A}}$) and retrograde ($J_{{PMJ}_{R}}$) propagation, respectively.

However, in the case of a Purkinje control volume, we have to consider three different possible configurations in our Purkinje networks (normal, branching, or terminal) as shown in Fig. 1. The total flux given by equation (11) is:

14 $$\begin{aligned} \underline{Normal:} \quad J_{tot}&= J_{x_{i+1/2}} - J_{x_{i-1/2}}, \end{aligned}$$

15 $$\begin{aligned} \underline{Branching:} \quad J_{tot}&= \sum _{j=1}^{N_{biff}} J_{x_{j}} - J_{x_{i-1/2}}, \end{aligned}$$

16 $$\begin{aligned} \underline{Terminal:} \quad J_{tot}&= J_{PMJ} - J_{x_{i-1/2}}, \end{aligned}$$

where $N_{biff}$ is the number of Purkinje control volumes linked to the bifurcation, and $J_{PMJ}$ is the flux associated at the Purkinje-muscle-junctions. Following a similar approach to the three-dimensional case, the fluxes $J_{x_{i+1/2}}$, $J_{x_{i-1/2}}$, and $J_{x_{j}}$ are calculated via finite differences at timestep $n+1$ alongside the Purkinje conductivity $\sigma _P$ associated to the surface of the discretised Purkinje control volume using harmonic means.

### Purkinje–myocardium coupling

To model the coupling between Purkinje–myocardium domains, we consider an additional flux $J_{PMJ}$. The electrical stimulus coming from the Purkinje system reaches the myocardium at specialised sites called Purkinje-muscle junctions (PMJs), spreading in the endocardium by a distance of approximately 1 *mm* between each other^33^. Importantly, PMJs are known to exhibit a characteristic asymmetric conduction delay due to electrotonic interactions of around 4–14 ms on the anterograde direction (Purkinje-to-myocardium)^34^ , and of about 2–4 ms when propagation occurs in the retrograde direction (myocardium-to-Purkinje)^34^. This behaviour is characterised as a source-sink mismatch phenomenon since a single Purkinje terminal may need to activate a bulk of myocardium tissue^35,36^.

Typically, the PMJ coupling is modelled by a fixed resistance, linking a Purkinje element to several myocardium elements^37^. We follow this approach by modelling the flux $J_{PMJ}~(\mu A/cm^{2})$ using a fixed resistance $R_{PMJ}$ and by coupling a single Purkinje control volume to its $N_{PMJ}$ closest myocardium control volumes, as shown in Fig. 1D. Moreover, the PMJ flux is given as a non-homogeneous Neumann boundary condition by:

17 $$\begin{aligned} J_{PMJ} = \frac{1}{h^{2}_{P}} \sum _{k=1}^{N_{PMJ}} \frac{ (V_{P} - V_{{M}_{k}}) }{R_{PMJ}}, \end{aligned}$$

where the sign of the flux determines if $J_{PMJ}$ exerts its action in the anterograde or retrograde direction (see Fig. 1E).

### Numerical scheme

For the iterative solution of the coupled model, we start by solving the reaction terms describing the Purkinje and myocardium cellular models, given by the non-linear systems of ODEs in Eqs. (7)–(8). We consider here the forward Euler method for simplicity, albeit monoalg3d is equipped with more advanced ODE schemes (such as Rush-Larsen and adaptive forward Euler). This gives:

18 $$\begin{aligned} C_{m} \frac{V_{d}^{n+1/2}-V_{d}^{n}}{\Delta t}&= \left[ -I_{{ion}_d}(V_{d}^{n},\eta _{d}^{n}) + I_{{stim}_d} \right] , \end{aligned}$$

19 $$\begin{aligned} \frac{\eta _{d}^{n+1/2} - \eta _{d}^{n}}{\Delta t}&= f_d (V_{d}^{n},\eta _{d}^{n}). \end{aligned}$$

PMJ fluxes are computed next based on Eq. (17), as:

20 $$\begin{aligned} J_{PMJ}^{n+1/2} = \frac{1}{h^{2}_{P}} \sum _{k=1}^{N_{PMJ}} \frac{ \left( V_{P}^{n+1/2} - V_{{M}_{k} }^{n+1/2} \right) }{R_{PMJ}}. \end{aligned}$$

The diffusion terms of the Purkinje and myocardium domains involves the solution of the linear system:

21 $$\begin{aligned} \beta C_m \frac{V_{d}^{n+1}-V_{d}^{n+1/2}}{\Delta t} = \nabla \cdot (\mathbf {\sigma _d} \nabla V_{d}^{n+1}) + J_{PMJ}^{n+1/2} h^{2}_{P}. \end{aligned}$$

Observe that by computing $J_{PMJ}$ at time $n+1/2$, we decouple the Purkinje and Myocardium domains. This enhances the solver’s modularity, allowing different classes to be used for each domain, but at the cost of numerical stability. While a fully implicit solution of the PDE is unconditionally stable, decoupling the two domains results in a conditionally stable scheme, where $R_{PMJ}$ constrains the maximum time step.

### ECG calculations

An approximation for the ECG can be computed by assuming that the tissue is immersed in an unbounded volume conductor^38^. The surface potential can be then calculated using the equation:

22 $$\begin{aligned} \phi _e = \dfrac{1}{4 \pi \sigma _{b}} \int _{\Omega } \dfrac{\beta I_{m}}{\Vert \vec {r} \Vert } d \Omega , \end{aligned}$$

where $\sigma _{b}$ is the bath conductivity, and $\vec {r}$ is the distance vector between source and field points, the latter essentially the electrode positions of the virtual ECG leads. The source term $\beta I_{m}$ is given by the solution of the diffusive term $\nabla \cdot (\sigma \nabla V_{m})$, which is available in every timestep.

To efficiently implement this new functionality in monoalg3d, we implemented the calculations of the Eq. (22) using OpenMP in CPUs or CUDA on GPUs environments.

### Performance efficiency strategies

#### Solving diffusion on GPUs

In previous work^28^, the linear system linked to the diffusion term in Eq. (9) was exclusively solved in the CPU using an OpenMP version of the conjugate gradient (CG) method. To enable the solution of large linear systems on GPUs, we first converted its sparse matrix representation from the ALG format to a Compressed Sparse Row (CSR) data structure compatible with the cuSparse library. This allows to directly solve the CG on the GPU by using this data structure together with the methods implemented in the cuBLAS library. It is worth noting that the biconjugate gradient (BCG) method is also available in this new version.

#### New output format

To minimise disk space usage and improve output performance, we provide novel support for EnSight files as new output format. This format is also compatible with multiple visualisation tools, such as Paraview, allowing most post-processing workflows to be kept unchanged.

#### MPI batch

Finally, for sensitivity analysis and uncertainty quantification studies, monoalg3d provides a novel feature for the concurrent dispatch of multiple simulations using the message passing interface (MPI) standard. Given a baseline simulation and a range of parameters, the solver generates automated configuration files for all possible combinations of input parameters. Each configuration file is then dispatched in parallel using MPI. This allows to upscale more efficiently the number of jobs running in HPC environments, enabling for instance to perform hundreds of simultaneous simulations for a given patient using a wide range of parameters. Such a feature is an important step towards in silico trials, drug therapy, and risk assessment studies.

### Computational simulations

**Fig. 2:**
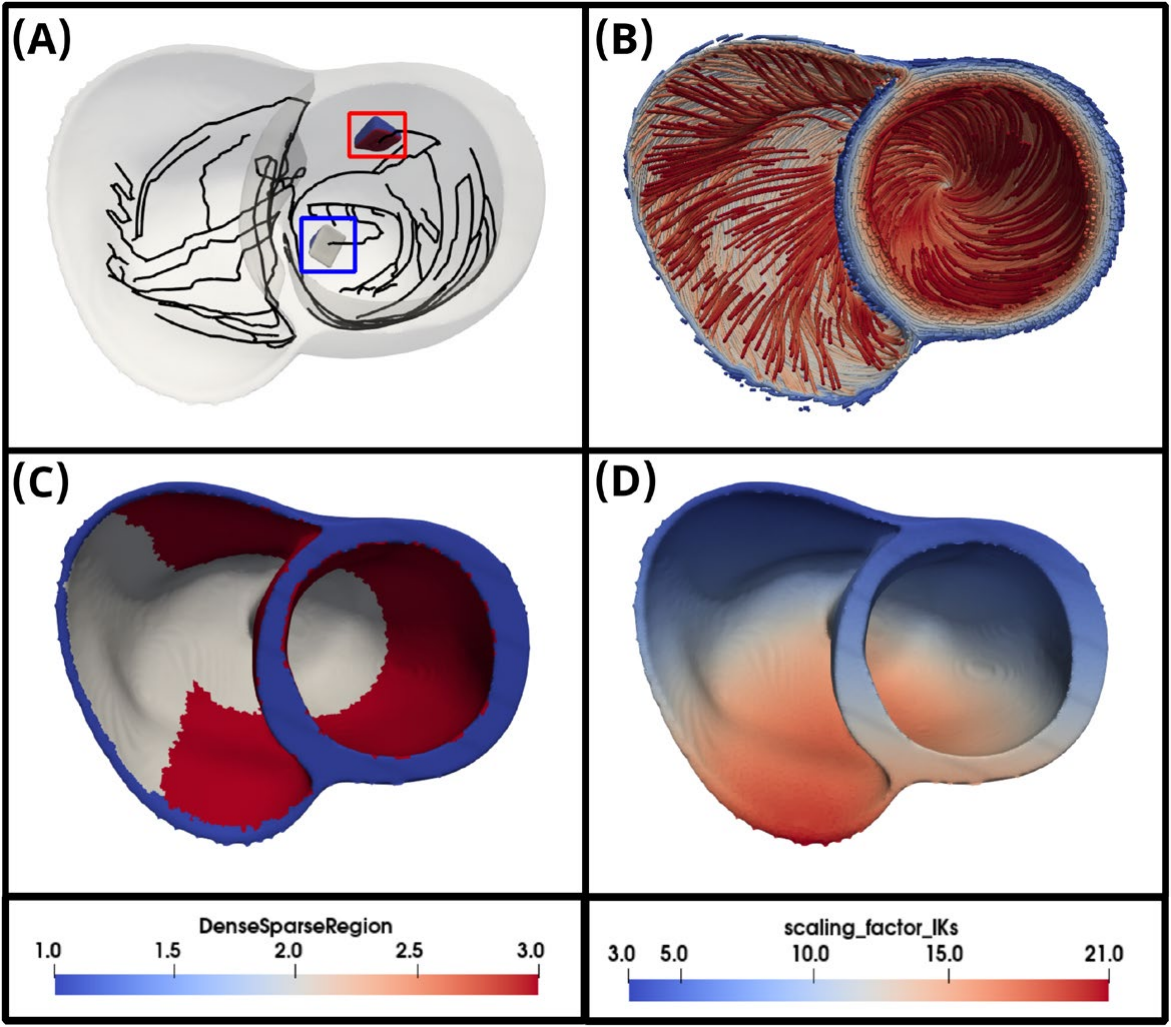
Human-based biventricular mesh used for patient-specific simulations. (**A**) Biventricular mesh coupled to the Purkinje network generated with the extra branching procedure from^26^. The Purkinje network is coloured in black and ventricular wedges used for the Purkinje coupling calibration are highlighted by red and blue squares. (**B**) Fibre orientation field of vector $\vec {f}$. (**C**) Heterogeneity in conductivity tensor $\sigma _M$, where blue denotes fully orthotropic conductivity values ($\sigma _f,\sigma _t,\sigma _n$), while white and red represent dense and sparse endocardial regions with isotropic conductivity values of $\sigma _{dense}$ and $\sigma _{sparse}$, respectively. (**D**) Scaling factor map of $I_{Ks}$ current in the *ToR-ORd* model used for T-wave personalisation^46^. This figure was generated using the last version of monoalg3d (https://github.com/rsachetto/MonoAlg3D_C) and visualised with the Paraview tool version 5.13.2.

Two sets of experiments were used to evaluate the improvements implemented in monoalg3d: a benchmark cuboid mesh to quantify performance improvements; a patient-specific mesh coupled to a Purkinje network (see Fig. 2), as an exemplar of application towards cardiac digital twinning.

All our numerical experiments were performed on the Polaris supercomputer provided by the Argonne Leadership Computing Facility, a 560 node HPE Apollo 6500 Gen 10+ system. Each computing node is equipped with a 2.8 GHz AMD EPYC Milan 7543P 32 core CPU with 512 GB of DDR4 RAM and four NVIDIA A100 GPUs with 40,960 *megabytes* of memory each.

#### Benchmark myocardium cuboid

A numerical test, adapted from Niederer et al.^39^, was conducted with minor domain size modifications to: (1) evaluate GPU speedups in solving non-linear ODEs and the parabolic PDE; (2) assess space adaptivity effects on efficiency; and (3) compare disk space usage between EnSight and VTK formats.

The test used a $1 \times 1 \times 1~cm^3$ myocardium cuboid with transverse anisotropic conduction $\sigma _{\parallel } = 1.334~mS/cm$, $\sigma _{\perp } = 0.176~mS/cm$), monodomain parameters $\beta =1400~cm^{-1}$, $C_{m}=1~\mu F/cm^{2}$, and human-based ventricular models: *ten Tusscher* (12 state variables)^40^ and *ToR-ORd* (43 state variables)^41^. Stimulation was applied in a $0.15 \times 0.15 \times 0.15~cm$ region for 2 *ms* at 53 *pA*/*pF*.

The nonlinear ODEs were solved using the Rush-Larsen scheme^42^ with $\Delta t=0.01~ms$, while the parabolic PDE used $\Delta t=0.02~ms$ for a total of 1500 ms. Without space adaptivity, uniform discretisation at $h_{M} = 250~\upmu m$ led to 64, 000 control volumes. Adaptive resolutions ranged from $h_{{M}_{min}} = 250~\upmu m$ to $h_{{M}_{max}} = 500~\upmu m$, with refinement/de-refinement bounds at 10.01/10.00 and adaptation every 10 timesteps.

The benchmark was tested across eight CPU/GPU configurations:

- A+OC+PC: Adaptive, ODEs on CPU, PDE on CPU;
- A+OG+PC: Adaptive, ODEs on GPU, PDE on CPU;
- A+OC+PG: Adaptive, ODEs on CPU, PDE on GPU;
- A+OG+PG: Adaptive, ODEs on GPU, PDE on GPU;
- OC+PC: Non-adaptive, ODEs on CPU, PDE on CPU;
- OC+PG: Non-adaptive, ODEs on CPU, PDE on GPU;
- OG+PC: Non-adaptive, ODEs on GPU, PDE on CPU;
- OG+PG: Non-adaptive, ODEs on GPU, PDE on GPU.

Mesh geometry and transmembrane potential were saved every 100 timesteps. More details on the benchmark setup are provided in Supplementary Fig. S3 section A.2.

#### Patient-specific model with Purkinje network

To demonstrate the full capabilities of the proposed GPU cardiac solver, we conducted a simulation study within a patient-specific biventricular model incorporating a Purkinje network. The study had three objectives: (1) evaluate solver performance in realistic scenarios; (2) calibrate Purkinje coupling parameters $R_{PMJ}$ and $N_{PMJ}$ to physiological anterograde PMJ delays; and (3) verify solver scalability for concurrent GPU simulations.

We used a human biventricular mesh (76-year-old female, 87 kg, $107~cm^{3}$ volume) reconstructed from MRI^43^, previously applied in clinical ECG personalization^44–46^. Figure 2, presents further anatomical details, including its Purkinje network coupling (Fig. 2A), fiber orientation field (Fig. 2B), subendocardial Purkinje coupling layers (Fig. 2C), and $I_{Ks}$ scaling factor map for T-wave personalization^45^ (Fig. 2D).

For cellular electrophysiology, we used the *ToR-ORd* human-based ventricular model^41^ with modifications for T-wave personalization^46^: 50% $I_{Kr}$ scaling, 5$\times$ $I_{Ks}$ scaling^47^, and reducing $\tau _{jca}$ from 75 to 60 *ms*. The Purkinje domain was modeled with the human-based Purkinje *Trovato* model^48^. Both ODE systems were solved via the Rush-Larsen scheme with $\Delta t = 0.01~ms$. PDEs used the same discretisation step for a total simulation time of 600 *ms*. The stimulus protocol consisted of a single pulse applied at the His bundle ($N_{cells} = 25$) with 40 *pA*/*pF* amplitude and 2 *ms* duration. For more details about the mesh configuration refer to Supplementary Material sections A.3 and A.4.

#### Purkinje-muscle-junctions calibration

To validate the Purkinje module and enable a physiological range for its coupling parameters, an initial PMJ calibration experiment was conducted using two ventricular wedges from the considered biventricular mesh, as highlighted in Fig. 2A. Both wedges are activated by a single Purkinje terminal and are located at different regions of the left ventricle (see Fig. 2A), in order to analyse the effects of the isotropic endocardial conductivities ($\sigma _{dense}$ and $\sigma _{sparse}$) on the anterograde PMJ delay. As so, the first wedge is located within the sparse endocardial region, while the second is in the dense endocardial region. Different space discretisations were also tested for both the Purkinje (100 and $250~\upmu m$) and the myocardial (250 and $500~\upmu m$) domains. Conductivities were calibrated using monodomain cable simulations to match conduction velocities (CVs) with an automatic parametrization strategy^49^, as summarised in Supplementary Table S3 (Supplementary Material section A.4). For the Purkinje coupling parameters, we considered a range of 25 equispaced values in $[100,2500]~k \Omega$ for the PMJ resistance, $R_{PMJ}$, and 10 equispaced values in [10, 100] for the number of myocardium control volumes linked to a terminal Purkinje control volume, $N_{PMJ}$.

Altogether, we performed 2000 simulations considering all possible combinations of wedges, space discretisations, and Purkinje coupling parameters. Simulations were executed for a total time of 50 ms, as we were only interested in measuring anterograde PMJ delays. These took $\approx 30~s$ to run enabled by the OG+PG setup. The PMJ delay was calculated as the time difference between the terminal Purkinje control volume and the closest myocardium control volume linked to it reaching a transmembrane potential threshold of $-40~mV$.

#### Large-scale biventricular simulations

To evaluate performance, we tested two myocardium space discretisations: a *coarse* mesh ($h_M=500~\upmu m$) with 855, 670 control volumes and a *fine* mesh ($h_M=250~\mu m$) with 6, 845, 360 volumes. The Purkinje domain used a fixed $h_P=250~\upmu m$ with 7, 948 volumes. The control volumes for the *fine* and *coarse* meshes are regular hexahedrons with fixed space discretisation $h_M$. Similarly, the control volumes of the Purkinje network are also represented as regular hexahedrons with a fixed space discretisation $h_P$. Solver scalability was assessed across three simulation setups:

- 1N1S: 1 node, 1 simulation;
- 1N4S: 1 node, 4 concurrent simulations;
- 128N512S: 128 nodes, 512 concurrent simulations.

A large-scale simulation study calibrated $R_{PMJ}$ and $N_{PMJ}$ for the full biventricular case within a physiological range using monoalg3d’s MPI batch processing (see Supplementary Fig. S4 in Supplementary Material section A.5). We ran 512 concurrent simulations, varying $R_{PMJ}$ and $N_{PMJ}$ based on the initial Purkinje-muscle-junctions calibration simulations over the ventricular wedges. It is important to highlight that for each concurrent biventricular simulation 8 CPU cores and 1 GPU are utilized, as this setup demonstrated the best performance. For the *coarse* mesh, $R_{PMJ}$ spanned $[100,1300]~k\Omega$ (32 values) and $N_{PMJ}$ [15, 50] (16 values). For the *fine* mesh, $R_{PMJ}$ ranged from $[500,2300]~k\Omega$. ECG comparison with clinical data was performed using Pearson’s correlation coefficient across all 8 leads (I, II, V1–V6).

## Results and discussion

### Myocardium cuboid benchmark

**Fig. 3:**
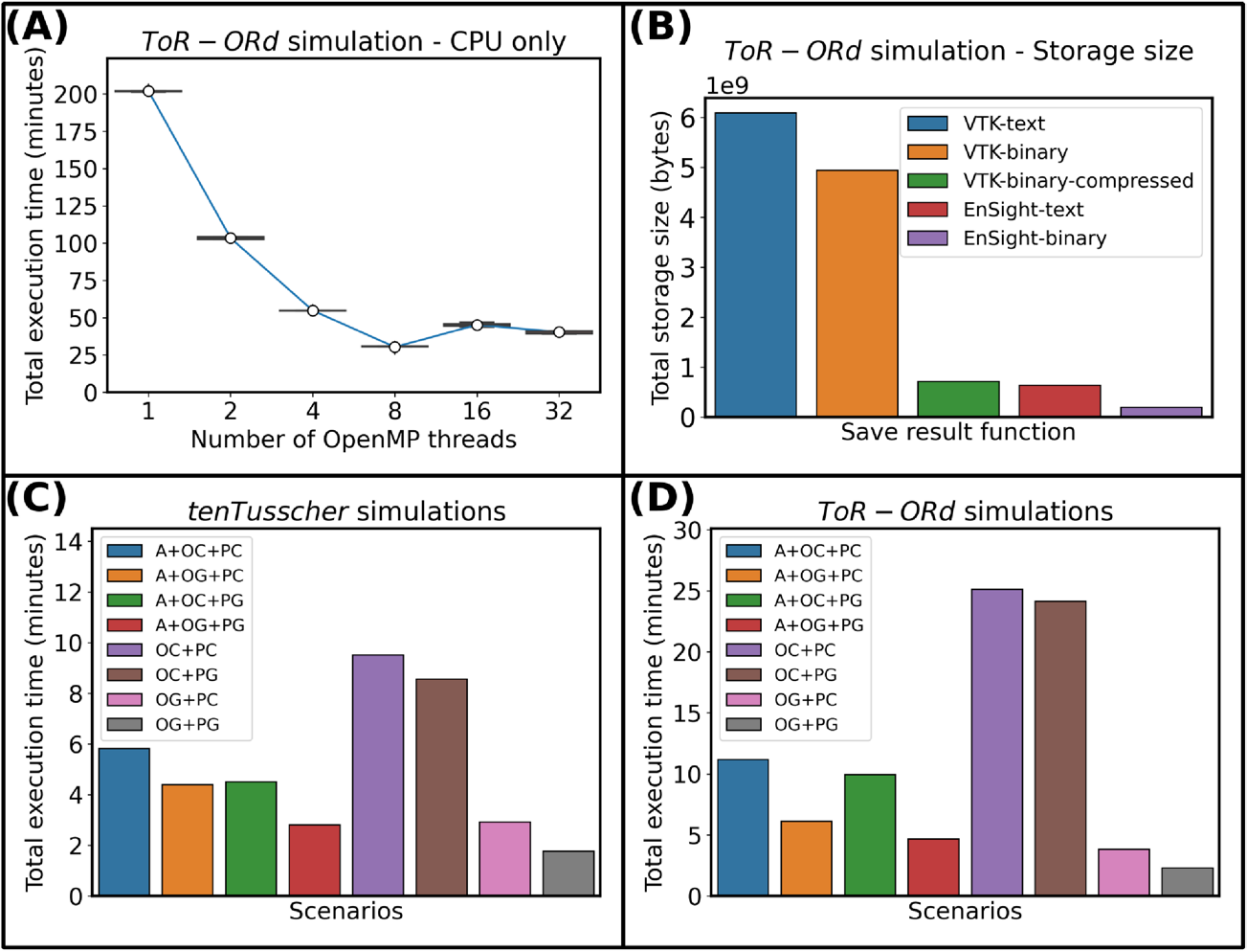
Results for the myocardium cuboid benchmark. (**A**) Execution time of the *ToR-ORd* simulation considering an OC+PC solver. (**B**) Disk usage for storing the mesh geometry and with the myocardial transmembrane potential for the *ToR-ORd* simulation using different file formats. (**C**) Total execution time for each of the 8 scenarios when using the *ten Tusscher* model. (**D**) Total execution time for each of the 8 scenarios with the *ToR-ORd* model.

An initial test was conducted using the OC+PC configuration, which uses entirely the CPU without space adaptivity to solve both the ODE and PDE systems, to evaluate the optimum number of OpenMP threads for the selected HPC facility. Five simulations were executed per number of threads, considering the human ventricular cellular *ToR-ORd* model (Fig. 3A). The best total execution times were found for an optimal number of 8 OpenMP threads, leading to a $\approx 6.66\times$ efficiency speedup, and enabling benchmark execution times around 30 min.

Similarly, input/output efficiency was optimised by considering 5 broadly adopted scientific formats: VTK-text (ASCII), VTK-binary, VTK-binary-compressed, EnSight-text (ASCII), and EnSight-binary (Fig. 3B). The results from this analysis highlight substantial savings in output file size when saving each model state variable (transmembrane potential in our case) in EnSight-binary format. This resulted in file storage sizes of merely 0.19 *gigabytes*, while VTK-text required around 6 *gigabytes* to store the same outputs. Therefore, the EnSight-binary can save approximately $31\times$, $25\times$, $3.5\times$ and $3.23\times$ more disk space when compared to VTK-text, VTK-binary, VTK-binary-compressed, and EnSight-text formats, respectively.

We then evaluated the solver’s performance for each of the 8 considered combinations of CPU/GPU architectures (see section *Computational simulations/Benchmark myocardium cuboid*), using either the *ten Tusscher* (Fig. 3C) or the *ToR-ORd* (Fig. 3D) cellular models. Based on the analysis above, 8 OpenMP threads were used in all the cases. The efficiency results presented in Fig. 3C for the *ten Tusscher* model yielded a maximum simulation time of $\approx 9~min$ for the OC+PC scenario (i.e., solving the entire problem in the CPU, without space adaptivity). Space adaptivity allowed the problem to be solved under 6 min in the CPU (A+OC+PC scenario), and in less than 3 min if both problems are solved in the GPU (A+OG+PG scenario). The largest efficiency improvement was however found when the problem was solved entirely in the GPU without space adaptivity (OG+PG scenario), decreasing the simulation time under 2 min. Equivalent results are presented in Fig. 3D for the *ToR-ORd* model for all the CPU/GPU configurations. Solving the simulation entirely on the CPU without space adaptivity (OC+PC scenario) yielded the most demanding execution time of $\approx 25~min$, while a $10.94\times$ efficiency gain and a total simulation time below 3 min were attained by exploiting the full GPU implementation (OG+PG scenario).

The results above indicate that space adaptivity (scenarios A+OC+PC, A+OG+PC, A+OC+PG and A+OG+PG) did not lead to any improvements in performance when compared to solving the fully refined mesh entirely on the GPU (scenario OG+PG). This behaviour can be attributed to the computational overhead associated with spatial adaptivity, specifically reassembling the matrix of the PDE and updating the grid data structures. In contrast, preloading the fully refined mesh and transferring all the data structures for both the ODEs and PDE to the GPU at the onset of a simulation offers significant advantages in terms of memory usage and computational performance when a fixed spatial discretisation is used. Therefore, the *ten Tusscher* model consumed around 436 megabytes of GPU memory when solved with the OG+PG scenario, while the *ToR-ORd* approximately 452 megabytes. This approach not only reduces data transfer between the host and the GPU, but also minimises memory allocation operations. Consequently, the computation at each time step is more regular than when space adaptivity is used, leading to an improved overall performance.

In addition, the joint analysis of the two considered cellular models revealed that solving the ODE system on the GPU was responsible for most of the performance gains. This becomes apparent by comparing scenarios OC+PC and OC+PG. For the *ten Tusscher* model, scenario OC+PG is just $1.11\times$ faster than scenario OC+PC, while for *ToR-ORd* a speedup of $1.04\times$ is obtained. However, when the ODE system was solved on the GPU (scenario OG+PC), a $3.26\times$ gain was attained for the *ten Tusscher* model when compared to scenario OC+PC, while this improvement was even more pronounced for *ToR-ORd*, and around $6.60\times$ gain. This result is primarily underlain by differences in algebraic complexity between both cellular models: the *ten Tusscher* model consists of 12 state variables, compared to 43 in *ToR-ORd*. Additionally, the *ToR-ORd* model involves a greater number of algebraic expressions, making it well-suited for GPU-based computations. Detailed execution times for both models can be found in the Supplementary Tables S1 and S2 section A.2. Finally, based on the convergence analysis for the cuboid benchmark executed using the latest versions of monoalg3d and opencarp and presented in the Supplementary Material section A.7, we verified that space discretisations of $500~\upmu m$ and $250~\upmu m$ provide relative errors below 220% and 45%, respectively, on both solvers when compared to a more refined mesh resolution of $50~\upmu m$ (see Supplementary Fig. S6). Therefore, spatial discretisation above $250~\upmu m$ should be avoided, especially under pathological scenarios, and the usage of a mesh resolution of $500~\upmu m$ for the biventricular mesh was purely motivated to demonstrate scalability of monoalg3d in the present work.

### Purkinje-muscle-junctions calibration reveals that anterograde delay is affected by different factors

**Fig. 4:**
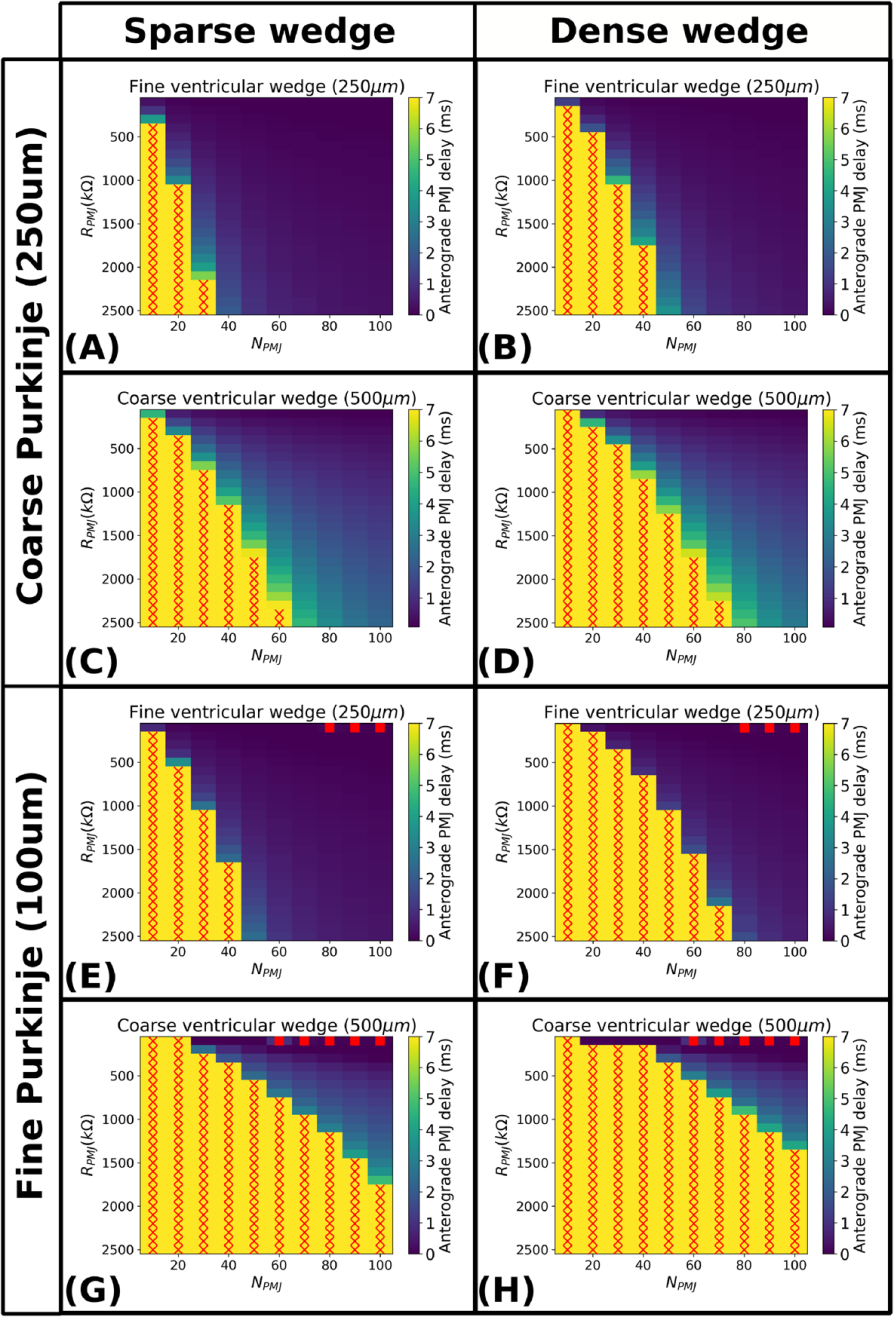
Heatmaps of anterograde PMJ delays for varying Purkinje coupling parameters $R_{PMJ}$ and $N_{PMJ}$ at different space discretisations. Red crosses denote propagation block at the PMJ site, while red squares denote instabilities associated to solving the Purkinje coupling explicitly. (**A**) Sparse endocardial region at fine discretisation, coupled to a coarse Purkinje fibre. (**B**) Dense endocardial region at fine discretisation, coupled to a coarse Purkinje fibre. (**C**) Sparse endocardial region at coarse discretisation, coupled to a coarse Purkinje fibre. (**D**) Dense endocardial region at coarse discretisation, coupled to a coarse Purkinje fibre. (**E**) Sparse endocardial region at fine discretisation, coupled to a fine Purkinje fibre. (**F**) Dense endocardial region at fine discretisation, coupled to a fine Purkinje fibre. (**G**) Sparse endocardial region at coarse discretisation, coupled to a fine Purkinje fibre. (H) Dense endocardial region at coarse discretisation, coupled to a fine Purkinje fibre. Fine spatial discretisation: $h_M=250~\mu m$, $h_P=500~\upmu m$. Coarse spatial discretisation: $h_M=500~\upmu m$, $h_P=250~\upmu m$. Conductivity values as per Supplementary Table S3 (Supplementary Material section A.4).

Figure 4 summarises how different factors affect the anterograde PMJ delay. First, refining the myocardial mesh reduces the delay, as the PMJ volume shrinks, we decrease the source-sink mismatch^36^. This effect is observable in pairwise comparisons such as Figures 4A–C, B–D, E–G, and F–H.

Second, Purkinje mesh resolution also influences the delay. Finer discretisation increases the delay due to reduced current per control volume (less source). Conversely, coarser meshes with larger Purkinje control volumes, deliver more current through the coupling interface, accelerating myocardial depolarisation. This trend appears in Fig. 4A–E, B–F, C–G, and D–H.

Third, endocardial conductivity impacts the delay, with denser regions, i.e., high conductivity regions, producing longer delays (Fig. 4A,B). Higher conductivity leads to greater current dispersion before threshold is reached, delaying activation. Similar effects appear in comparisons Fig. 4C,D,E,F,G,H. In addition, we report some instabilities (red squares in Fig. 4) due to the PMJ coupling being solved explicitly in Eq. (20).

Lastly, anterograde PMJ delay depends on coupling parameters. Increasing $R_{PMJ}$ raises the delay and may even block propagation (red crosses in Fig. 4) due to reduced current flow. In contrast, increasing $N_{PMJ}$, the number of coupled myocardial volumes, lowers the delay by stimulating larger regions of the myocardium.

### Large-scale biventricular simulations with anterograde PMJ delay calibration are a step towards cardiac digital twinning

An initial simulation that varied the number of OpenMP threads using the OG+PG scenario was executed to verify the optimal number of threads to be used for the *coarse* and *fine* meshes. Based on the results in Table 1, all patient-specific simulations were executed entirely on the GPU using 8 OpenMP threads, exploiting one GPU device for both the Purkinje and myocardium domains as this configuration demonstrated the best performance.

**Table 1:** Execution times (minutes) for the patient-specific simulations using the OG+PG scenario with a varying number of OpenMP threads.

	OpenMP threads					
Mesh	1	2	4	8	16	32
*coarse* ($h_M=500~\upmu m$)	44.93	29.49	19.00	**14.95**	13.61	13.43
*fine* ($h_M=250~\upmu m$)	371.96	269.51	225.55	**223.93**	235.22	235.88

OG+PG: Non-adaptive, ODEs on GPU, PDE on GPU.

**Fig. 5:**
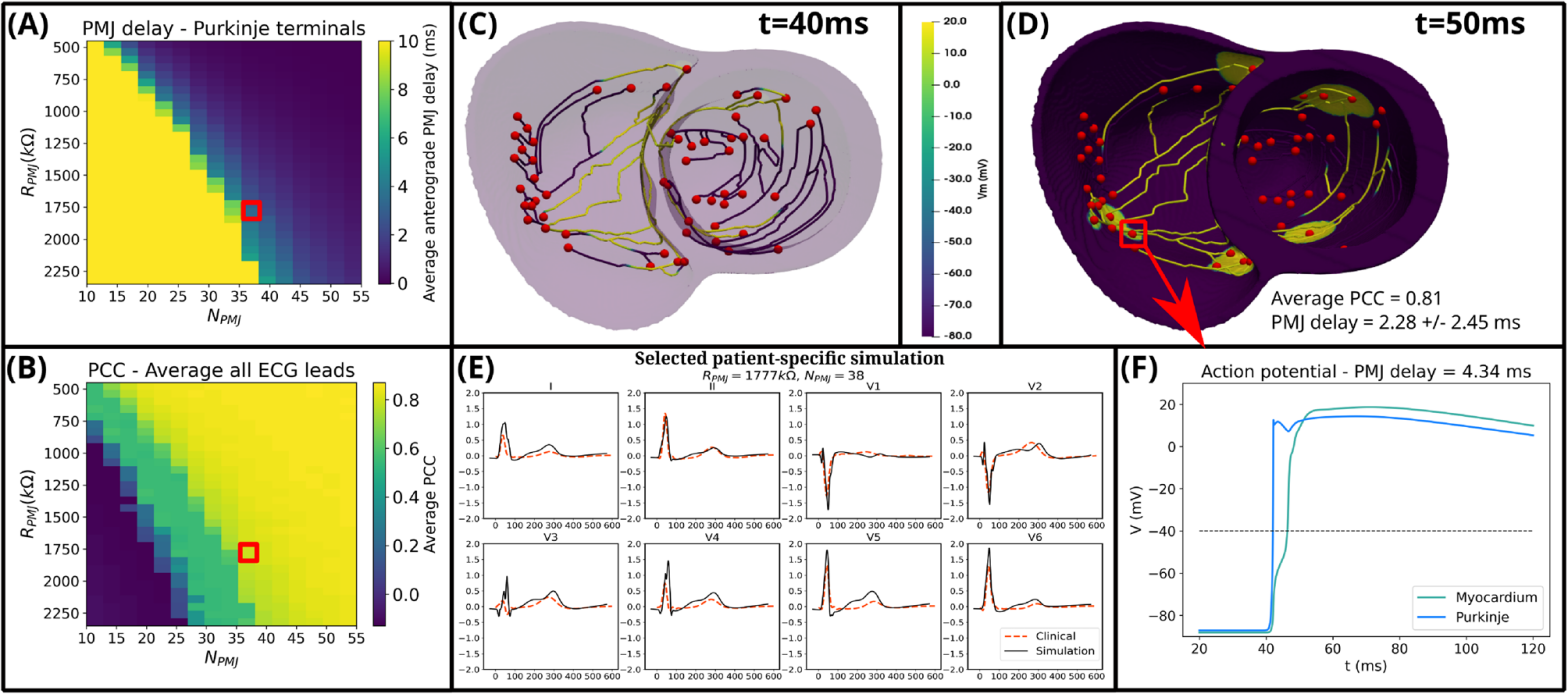
Results for the 512 patient-specific simulations with the *fine* mesh. (**A**) Average anterograde PMJ delay across all Purkinje terminals. (**B**) Average PCC across all leads between the clinical and simulated ECG. Selected Purkinje coupling parameters $(R_{PMJ}=1777~k \Omega ,N_{PMJ}=38)$ are highlighted by red squares. (**C,D**) Selected patient-specific simulation with an average PCC of 0.81 in ECG reconstruction and average anterograde PMJ delay of $2.28 \pm 2.45~ms$, at times $t=40$ and $t=50~ms$, respectively. (**E**) Comparison between clinical and simulated ECGs. (**F**) Action potential upstrokes for the PMJ site highlighted in panel (**D**) with anterograde PMJ delay of 4.34 *ms*, at the terminal Purkinje volume (blue) and its closest coupled myocardium volume (lime). The 3D models in panels (**C,D**) were generated using the last version of monoalg3d (https://github.com/rsachetto/MonoAlg3D_C) and visualised with the Paraview tool version 5.13.2.

Figure 5 illustrates selected simulation results at *fine* mesh resolution for the identified optimal set of Purkinje coupling parameters from a total of 512 executions. This set was chosen based on similarity between the simulated and clinical ECG signals across all leads, as well as replicating a range of physiological anterograde PMJ delays across all Purkinje terminals when activating the myocardium. Nevertheless, the existence of other combinations of Purkinje coupling parameters yielding a similar activation pattern (Fig. 5A,B) indicates that a range of cardiac digital twins can be approximated by the considered parameters, $R_{PMJ}$ and $N_{PMJ}$. Ventricular activation started $\approx 48~ms$ after His-bundle pacing (Fig. 5C,D), with the whole biventricular domain being activated in around 110 *ms* (end of simulated QRS complex, Fig. 5E), also within the physiological range of 80–120 ms for healthy subjects^33^. Moreover, the inclusion of heterogeneity in $I_{Ks}$ from^46^ generated a reasonable approximation for the T-wave (Fig. 5E), with an average PCC of 0.81 across all the 8 independent ECG leads (I, II, V1–V6). The results for the *coarse* mesh simulations are presented in the Supplementary Material section A.6 (see Supplementary Fig. S5).

The effects of anterograde PMJ delays were correctly recovered as shown in Fig. 5D, as well as in Fig. 5F for a PMJ site on the right ventricle. As it can be seen in the latter, the closest myocardial control volume to the Purkinje terminal could not be activated instantaneously, due to the source-sink mismatch between the Purkinje terminals and myocardial cells^35,36,50^. The myocardial control volume only became entirely depolarised approximately 4.34 ms after the stimulus reached the PMJ site, exhibiting an initial brief spike followed by a distinct blunted depolarisation (Fig. 5F). This behaviour, reported in different experimental studies^51,52^ and attributed to the electrotonic effects at the junctions.

In terms of scalability, Table 2 summarises execution times for our considered submission scenarios. For the 1N1S scenario (1 computing node, 1 simulation), total execution times for the *coarse* and *fine* discretisations were around 15 and 221 min, respectively. Similar execution times without significant performance loss were observed when 4 concurrent simulations were executed in the same compute node (scenario 1N4S), with average total times around 22 and 210 min for the *coarse* and *fine* meshes, respectively. A similar behaviour was observed using the MPI batch feature for the 128N512 scenario, with execution times of approximately 22 and 212 min for *coarse* and *fine* resolutions, respectively. Considering the times for the MPI process to start and end, these values were around 23 and 302 min, respectively. From the results in Table 2, it also transpires that the most demanding component is the solution of the ODE system of the myocardium, contributing around 11 and 94 min for the *coarse* and *fine* mesh resolutions, respectively. This is explained due to the larger number of control volumes of this domain compared to the Purkinje one, making this section of the problem more computationally demanding and the overall bottleneck.

In addition, to highlight the performance gains achieved with the novel GPU-based linear system, we ran the *coarse* mesh simulation using the OG+PC and OG+PG configurations with varying numbers of OpenMP threads. For the OG+PC scenario, the total execution time was approximately 191.45 min with 8 OpenMP threads and 96.14 min with 32 threads. In contrast, the OG+PG configuration significantly reduced the total runtime to around 14.97 min and 13.47 min for 8 and 32 threads, respectively. Focusing on the time spent solving the myocardium PDE system, OG+PC required 178.07 min and 83.39 min, whereas OG+PG completed the same task in just 4.25 min and 3.56 min, when using 8 and 32 threads, respectively. In terms of GPU memory consumption for the *coarse* and *fine* meshes, approximately 1146 *megabytes* and 5544 *megabytes* are allocated in a single GPU when solved with the OG+PG configuration.

**Table 2:** Execution times (minutes) for the patient-specific simulations for the 3 submission scenarios considering a total simulation time of $t_{max} = 600~ms$.

Mesh	Scenario	*Total*	*Write*	*ECG*	$M_{ODE}$	$M_{PDE}$	$P_{ODE}$	$P_{PDE}$	$MPI_{end}$
	1N1S	**15.15**	0.08	0.07	7.78	4.27	0.20	1.01	–
*coarse*	1N4S	**22.24 ± 0.21**	0.07 ± 0.00	0.11 ± 0.00	10.94 ± 0.11	7.23 ± 0.09	0.23 ± 0.00	1.05 ± 0.01	23.15
	128N512S	**22.41 ± 0.32**	0.07 ± 0.00	0.11 ± 0.02	11.06 ± 0.17	7.24 ± 0.15	0.23 ± 0.01	1.06 ± 0.01	23.55
	1N1S	**221.49**	0.52	0.42	97.62	87.70	0.26	1.13	–
*fine*	1N4S	**210.66 ± 0.32**	0.50 ± 0.00	0.42 ± 0.00	93.80 ± 0.18	84.81 ± 0.05	0.39 ± 0.01	1.13 ± 0.00	213.13
	128N512S	**211.94 ± 7.33**	0.49 ± 0.01	0.42 ± 0.00	93.87 ± 0.50	85.89 ± 5.56	0.37 ± 0.03	1.13 ± 0.01	302.29

For the *coarse* mesh the total time is around 15.15, 22.24 and 22.41 minutes for the 1N1S, 1N4S and 128N512S scenarios, respectively, while for the *fine* mesh the total time is approximately 221.49, 210.66 and 211.94 minutes for the same scenarios. *Total*: total time; *Write*: writing time; *ECG*: ECG computation time; $M_{ODE}$/$M_{PDE}$: time to solve the myocardium ODE/PDE system; $P_{ODE}$/$P_{PDE}$: time to solve the Purkinje ODE/PDE system; $MPI_{end}$: time for the MPI process to finish.

**Fig. 6:**
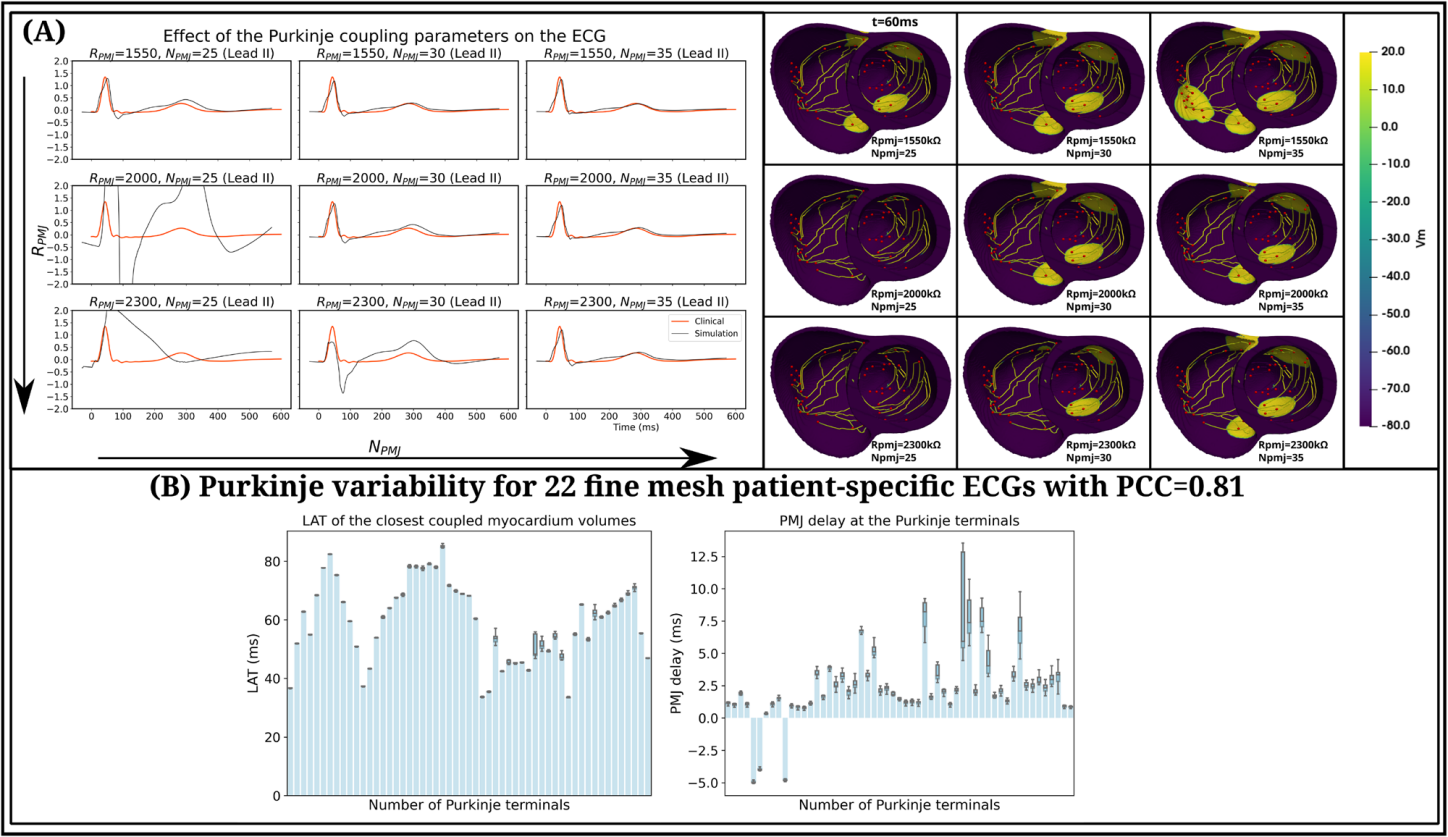
(**A**) Effects of Purkinje coupling parameters $R_{PMJ}$ and $N_{PMJ}$ on the simulated ECG (Lead II) and on biventricular activation, considering the *fine* mesh. The left panel illustrates 9 simulated ECGs (black traces) for different combinations of $R_{PMJ}$ and $N_{PMJ}$, compared against the clinical one (in red). The right panel illustrates the activation pattern at time $t=60~ms$ for each of the 9 corresponding simulations. (**B**) Comparison between 22 patient-specific simulations using the *fine* mesh and average PCC of 0.81 for different combinations of the Purkinje coupling parameters. Left panel, boxplots of local activation times (*ms*) from the closest coupled myocardial control volumes to each of the 55 terminals located in the generated Purkinje network, while in the right panel boxplots for the anterograde PMJ delay (*ms*) for each Purkinje terminal. The 3D models in panel (**A**) were generated using the last version of monoalg3d (https://github.com/rsachetto/MonoAlg3D_C) and visualised with the Paraview tool version 5.13.2.

Additional results on the performed patient-specific simulations are presented in Fig. 6, where 9 representative simulations using the *fine* mesh show how the Purkinje coupling parameters can impact ventricular activation. Furthermore, in Fig. 6A there is further evidence supporting the existence of multiple possible simulations with a similar ECG. These results also indicate that, while sustaining analogous ECGs, different combinations of Purkinje coupling parameters can generate distinct distributions of PMJ delays across the Purkinje terminals. Based on that, certain Purkinje terminals exhibit more variability in their associated PMJ delays, which might indicate a more important role of such PMJs to the whole ventricular activation.

Another interesting result is presented in Fig. 6B, which illustrates the results from 22 patient-specific simulations using the *fine* mesh, all with an average PCC of 0.81 for the ECG. The analysis of local activation times (LATs) from the closest coupled myocardium control volume to each Purkinje terminal also reveals that, in almost all terminals, the average LAT does not significantly change (see left panel Fig. 6B). This is however with the exception of a small number of Purkinje terminals, which present larger LAT variability of up to 9.11 ms. Moreover, the analysis of PMJ delays revealed an even greater variability across Purkinje terminals (see right panel Fig. 6B), while still producing a similar ECG morphology. These findings further support the concept of non-uniqueness in ventricular activation patterns, an observation recently highlighted by Gradits et al.^53^. This suggests that, for a given patient, multiple plausible cardiac digital twin configurations may exist.

Finally, another relevant finding from these simulations is the existence of an almost linear relation between the Purkinje coupling parameters and the appearance of propagation block, as analysed by Figs. 5A and S5A (Supplementary Material section A.6).

Based on these results, the novel MPI batch feature illustrates the scalability and efficiency of the proposed cardiac solver to conduct patient-specific studies under cluster GPU environments and is a step towards cardiac digital twinning, enabling the correct adjustment of sensitive Purkinje coupling parameters to physiological ranges for anterograde PMJ delays.

## Limitations

This study employed a regular voxel-based (cubic) discretisation of the cardiac geometry, which can introduces staircase approximations along curved surfaces. We acknowledge this geometric simplification; however, because our simulations are based on the monodomain model, a reaction–diffusion formulation for transmembrane potential and focus exclusively on activation propagation without extracellular stimulation, prior studies indicate that such surface approximations have only a modest influence on bulk conduction metrics^54,55^. To ensure robustness, a mesh-refinement analysis was performed, showing activation time variations below 5.56% for a mesh resolution of $100~\upmu m$, that can be verified in Supplementary Material section A.7. Therefore, we consider the staircase effect to have negligible impact on our main findings. Nevertheless, for applications involving extracellular potentials, defibrillation, or torso coupling, a boundary-fitted or smoothed mesh representation would be more appropriate.

## Conclusion

In this work, we presented an open-source, high-performance GPU-based solver for cardiac electrophysiology simulations. Our main objective was to enhance the efficiency and scalability of large-scale simulations while incorporating physiologically relevant features such as the Purkinje conduction system. Through systematic evaluation across diverse CPU/GPU configurations, we demonstrated that executing both ODE and PDE solvers on the GPU significantly accelerates computations, especially for complex human-based cellular models. Compared to our previous work, we found that transferring all required data structures to the GPU at initialization yields better performance than space adaptivity strategies. Additionally, patient-specific simulations with calibrated anterograde PMJ delays highlight the solver’s suitability for realistic digital twin applications. The solver also demonstrated excellent scalability, with the ability to run 512 simulations concurrently across 128 compute nodes, achieving per simulation execution times close to the one using a single node and with linear speedups in terms of throughput. This scalability, combined with the solver’s support for detailed conduction modelling and efficient output formats, positions it as a practical and accessible tool for large-scale in silico studies. To our knowledge, this is the first open-source solver to offer high-throughput simulations with detailed Purkinje-muscle coupling. These capabilities lay the foundation for digital twin frameworks and virtual patient cohorts, marking a step toward in silico clinical trials and personalized therapy evaluation.

## Supplementary Information

Supplementary Information.

## Data Availability

The source code for the open-source cardiac solver is publicly available at https://github.com/rsachetto/MonoAlg3D_C. All the necessary configuration files, custom functions, post-processing scripts used during the current study, as well as, the biventricular mesh and Purkinje networks used for the patient-specific simulations are publicly available at the Zenodo repository https://doi.org/10.5281/zenodo.15041476 to allow reproducibility.
