# Supplementary material for: Toward cardiac electrophysiology digital twins with an efficient open source scalable solver on GPU clusters
Source: Sci Rep. 2026 Feb 18;16:9619. doi: 10.1038/s41598-025-33709-w (PMC13009401; doi:10.1038/s41598-025-33709-w)
Supplement: Supplementary file 1 — Supplementary Information. [file 41598_2025_33709_MOESM1_ESM.pdf]

Supplementary Material

Toward Cardiac Electrophysiology Digital Twins with an Efficient Open Source Scalable Solver on GPU Clusters

Lucas Arantes Berg[+,*], Rafael Sachetto Oliveira[+,*], Julia Camps, Lucas Marins Ramalho de Lima, Joventino de Oliveira Campos, Zhinuo Jenny Wang, Ruben Doste, Alfonso Bueno-Orovio, Rodrigo Weber dos Santos[†], Blanca Rodriguez[†]

[+] **Lucas Arantes Berg and Rafael Sachetto Oliveira made equal contributions to this work.**
[*] **Lucas Arantes Berg and Rafael Sachetto Oliveira are the corresponding authors to this work.**
[†] **Blanca Rodriguez and Rodrigo Weber dos Santos are the senior authors to this work.**
Emails: lucas.arantesberg@cs.ox.ac.uk and sachetto@ufsj.edu.br

A Supplementary Material

A.1 Finite volume method applied to the anisotropic monodomain model

In this section we describe how the FVM is applied to solve the anisotropic monodomain model for the myocardium. As explained in the main text, the reaction and diffusion terms of the model can be separated using operator splitting. This leads, per timestep, to the solution of the parabolic linear PDE:

$$\beta C_m \frac{\partial V_M}{\partial t} = \nabla \cdot (\sigma_{\mathbf{M}} \nabla V_M). \tag{1}$$

For the spatial discretisation of its diffusion term, we consider the relations:

$$J = -\sigma \nabla V, \tag{2}$$

where J $(\mu A/cm^2)$ represents the density of intracellular current flow and

$$\nabla \cdot J = -I_v. \tag{3}$$

Here, I_v $(\mu A/cm^3)$ is regarded a volumetric membrane current and corresponds to the right side of equation (1).

After defining the mesh geometry and partitioning the domain in control volumes, the FVM equations can be written. Integrating equation (3) over each discretised volume:

$$\int_\Omega \nabla \cdot J \, dv = -\int_\Omega I_v \, dv, \tag{4}$$

and applying the divergence theorem together with the original PDE for the given domain, it yields:

$$\beta C_m \int_\Omega \frac{\partial V}{\partial t} dv = -\int_\Omega I_v \, dv = \int_\Omega \nabla \cdot J \, dv = \int_{\partial\Omega} J \cdot \vec{n} \, ds, \tag{5}$$

where $\vec{n}$ represents the surface normal vector. This equation is the basic term for deriving the linear system of equations associated with the discretisation of the PDE problem.

Let us consider for simplicity a bidimensional uniform mesh, consisting of regular squares with a space discretisation h_M. Situated in the centre of each volume (i, j) is a node, and the transmembrane potential V_M is the variable of interest. Assuming that I_v represents an averaged value in each square, and using the result from equation (10) from the main manuscript, we have:

$$\left(\beta C_m \frac{\partial V_M}{\partial t} \right) \Bigg|_{(i,j)} = \frac{-\int_{\partial\Omega_M} J_{i,j} \cdot \vec{n} \, ds}{h_M^2}. \tag{6}$$

The calculus of $J_{i,j}$ can be split as shown in Figure S1A as the sum of the flows on the four faces of the control volume:

$$\int_{\partial\Omega} J_{i,j} \cdot \vec{n} \, ds = (J_{x_{i+i/2,j}} - J_{x_{i-i/2,j}} + J_{y_{i,j+i/2}} - J_{y_{i,j-i/2}}) h_M, \tag{7}$$

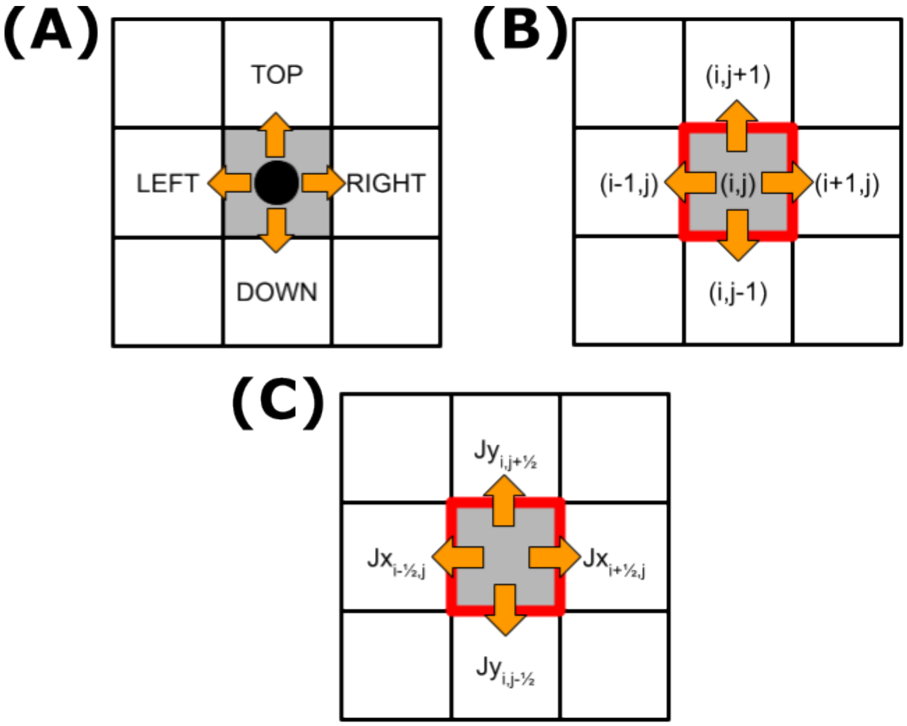

Figure S1 . Schematic of the 4 fluxes used to compute the intracellular current flow J for a control volume in the bidimensional anisotropic case.

where

$$\begin{bmatrix} J_x \\ J_y \end{bmatrix} = \begin{bmatrix} \sigma_x & \sigma_{xy} \\ \sigma_{xy} & \sigma_y \end{bmatrix} \cdot \begin{bmatrix} \frac{\partial V}{\partial x} \\ \frac{\partial V}{\partial y} \end{bmatrix}. \tag{8}$$

In terms of indexes, the mentioned flows passing through the faces of control volume (i,j) are given by $(i,j+1)$, $(i,j-1)$, $(i+1,j)$ and $(i-1,j)$, respectively, as depicted in Figure S1B. In addition, the fluxes through each face are shown in Figure S1C and are given by:

$$\begin{aligned} J_{x_{i\pm1/2,j}} &= \sigma_{x_{i\pm1/2,j}} \left.\frac{\partial V}{\partial x}\right|_{i\pm1/2,j} + \sigma_{xy_{i\pm1/2,j}} \left.\frac{\partial V}{\partial y}\right|_{i\pm1/2,j}, \\ J_{y_{i,j\pm1/2}} &= \sigma_{xy_{i,j\pm1/2}} \left.\frac{\partial V}{\partial x}\right|_{i,j\pm1/2} + \sigma_{y_{i,j\pm1/2}} \left.\frac{\partial V}{\partial y}\right|_{i,j\pm1/2}. \end{aligned} \tag{9}$$

To calculate the flows in equation (9), the conductivity tensor needs to be evaluated at the faces of the control volume using the average value between the two neighbouring volumes:

$$\begin{aligned} \sigma_{x_{i\pm1/2,j}} &= \frac{\sigma_{x_{i\pm1,j}} + \sigma_{x_{i,j}}}{2}, \\ \sigma_{y_{i,j\pm1/2}} &= \frac{\sigma_{y_{i,j\pm1}} + \sigma_{y_{i,j}}}{2}, \\ \sigma_{xy_{i\pm1/2,j}} &= \frac{\sigma_{xy_{i\pm1,j}} + \sigma_{xy_{i,j}}}{2}, \\ \sigma_{xy_{i,j\pm1/2}} &= \frac{\sigma_{xy_{i,j\pm1}} + \sigma_{xy_{i,j}}}{2}, \end{aligned} \tag{10}$$

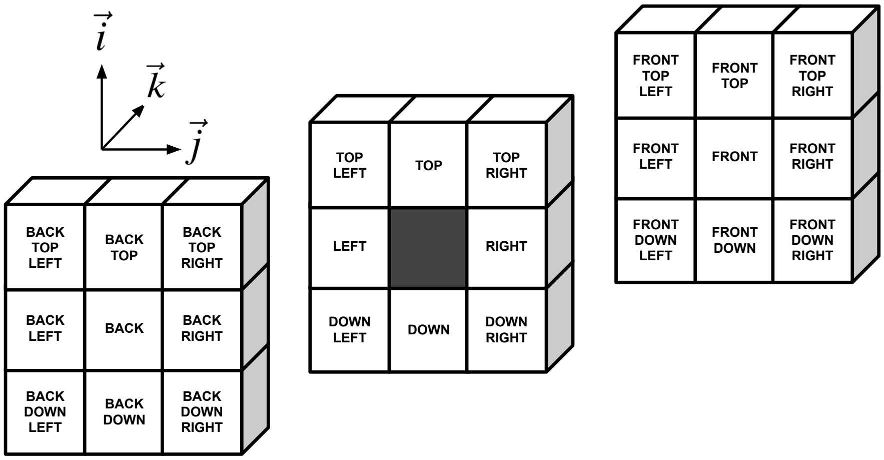

Figure S2 . Schematic of the 26 neighbourhood used to compute the intracellular current flow J in the tridimensional anisotropic case.

while the spatial derivatives are approximated using a finite difference approach:

$$\begin{aligned}
\left.\frac{\partial V}{\partial x}\right|_{i+1/2,j} &= \frac{(V_{i+1,j} - V_{i,j})}{h_M}, \\
\left.\frac{\partial V}{\partial x}\right|_{i-1/2,j} &= \frac{(V_{i,j} - V_{i-1,j})}{h_M}, \\
\left.\frac{\partial V}{\partial y}\right|_{i+1/2,j} &= \frac{(V_{i+1,j+1} + V_{i,j+1} - V_{i+1,j-1} - V_{i,j-1})}{4h_M}, \\
\left.\frac{\partial V}{\partial y}\right|_{i-1/2,j} &= \frac{(V_{i-1,j+1} + V_{i,j+1} - V_{i-1,j-1} - V_{i,j-1})}{4h_M}, \\
\left.\frac{\partial V}{\partial x}\right|_{i,j+1/2} &= \frac{(V_{i+1,j+1} + V_{i+1,j} - V_{i-1,j+1} - V_{i-1,j})}{4h_M}, \\
\left.\frac{\partial V}{\partial x}\right|_{i,j-1/2} &= \frac{(V_{i+1,j-1} + V_{i+1,j} - V_{i-1,j-1} - V_{i-1,j})}{4h_M}, \\
\left.\frac{\partial V}{\partial y}\right|_{i,j+1/2} &= \frac{(V_{i,j+1} - V_{i,j})}{h_M}, \\
\left.\frac{\partial V}{\partial y}\right|_{i,j-1/2} &= \frac{(V_{i,j} - V_{i,j-1})}{h_M}.
\end{aligned} \tag{11}$$

Similarly, for the tridimensional case, the equations follow an analogous pattern and can be derived using the same approach. The main differences are the inclusion in equation (7) of two additional flows in the z-axis ($J_{z_{i,j,k+1/2}}$ and $J_{z_{i,j,k-1/2}}$), and the application of a stencil considering all the 26 neighbouring control volumes, as shown in Figure S2.

A.2 Additional results for the cuboid benchmark

Figure S3 illustrates the propagation of excitation for the benchmark cuboid test (Figs. S3A-D), alongside corresponding action potentials from the *ten Tusscher* (Fig. S3E) and *ToR-ORd* (Fig. S3F) cellular models at the centre of the domain. Additionally, Figures S3G-H present the total number of control volumes to solve in the mesh over time when the space adaptivity was used for each model. As expected, when space adaptivity is active, the number of control volumes to be solved decreases as the domain starts to repolarise.

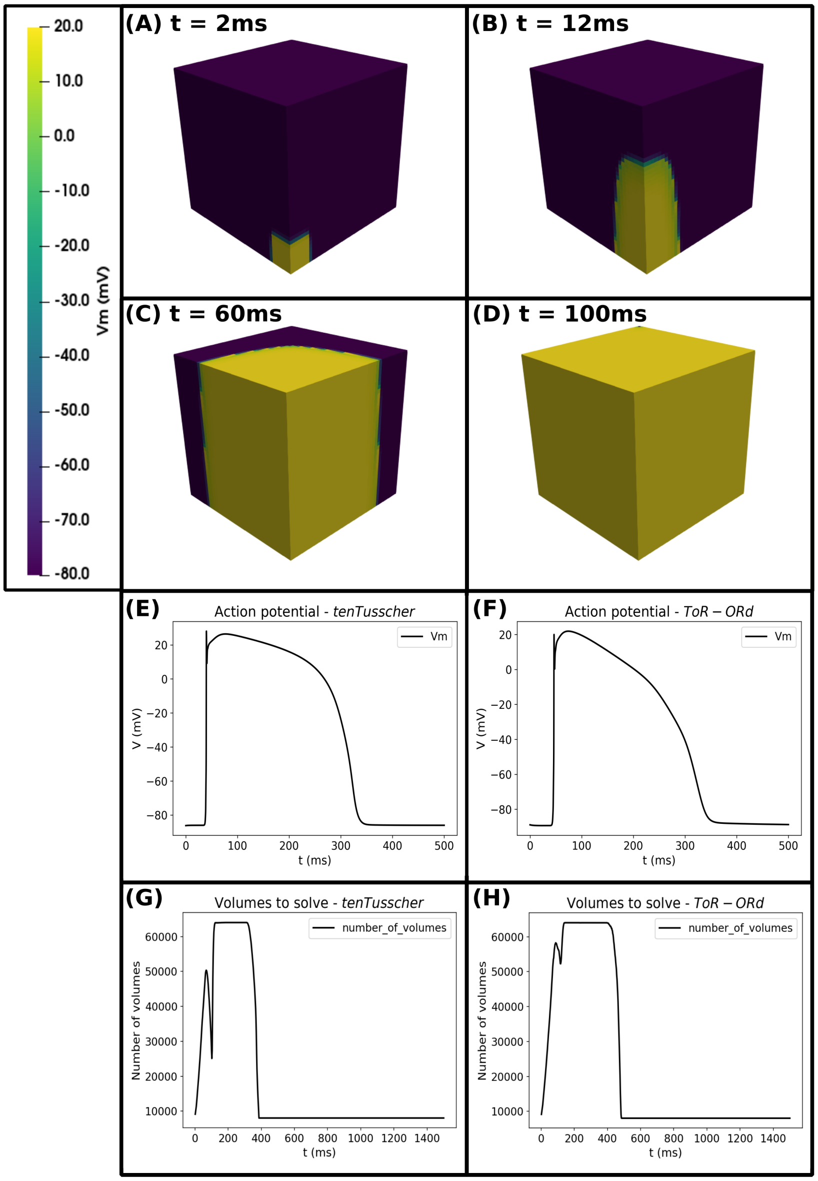

Figure S3 . Time evolution of transmembrane potential (V_M) for the benchmark cuboid test. (A-D) Snapshots at $t = 2\ ms$, $t = 12\ ms$, $t = 60\ ms$, and $t = 100\ ms$, respectively, when using the *ToR-ORd* cellular model. (E-F) Action potentials at the domain centre for the *ten Tusscher* and *ToR-ORd* cellular models, respectively. (G-H) Number of control volumes to solve in the mesh over time when using space adaptivity for both cellular models. The 3D models in panels (A-D) were generated using the last version of MonoAlg3D (`https://github.com/rsachetto/MonoAlg3D_C`) and visualised with the Paraview tool version 5.13.2.

Tables S1 and S2 summarise the execution times for all the 8 scenarios and the two cellular models considered. Times spent on each portion of the solver are also detailed.

A.3 Patient-specific mesh configuration

In these experiments, we use a human biventricular mesh (76 years old female, 87 kg, heart rate 48 bpm, 107 cm^3 volume), reconstructed from magnetic resonance imaging following[1]. This mesh has also been used in previous studies to personalise the subject's clinical ECG[2–4]. Further anatomical details are shown in Figure 2 of the main manuscript, including its coupling to the Purkinje network generated to activate the earliest activation sites inferred in[2,3] at given stimulation times (Fig. 2A), and the fibre orientation field of vector $\vec{f}$, equation (5) from the main manuscript (Fig. 2B). In addition, two thin layers are considered to

Table S1 . Execution times for the benchmark cuboid test in minutes (min) for the *ten Tusscher* cellular model in the 8 considered scenarios. A+OC+PC: Adaptive, ODEs on CPU, PDE on CPU; A+OG+PC: Adaptive, ODEs on GPU, PDE on CPU; A+OC+PG: Adaptive, ODEs on CPU, PDE on GPU; A+OG+PG: Adaptive, ODEs on GPU, PDE on GPU; OC+PC: Non-adaptive, ODEs on CPU, PDE on CPU; OC+PC: Non-adaptive, ODEs on CPU, PDE on CPU; OC+PG: Non-adaptive, ODEs on CPU, PDE on GPU; OG+PC: Non-adaptive, ODEs on GPU, PDE on CPU; OG+PG: Non-adaptive, ODEs on GPU, PDE on GPU.

Scenario	Total	Write	ODE	PDE	Reassemble matrix	Refine grid	Derefine grid	Order grid	Update cells	Update state vector
A+OC+PC	5.82	0.42	2.42	1.99	0.13	0.38	0.32	0.10	0.01	0.00
A+OG+PC	4.40	0.42	0.48	2.00	0.13	0.40	0.34	0.10	0.01	0.35
A+OC+PG	4.51	0.43	2.42	0.43	0.10	0.34	0.27	0.10	0.01	0.00
A+OG+PG	2.81	0.43	0.33	0.43	0.10	0.34	0.27	0.09	0.01	0.33
OC+PC	9.51	0.89	7.21	1.36	0.00	0.00	0.00	0.00	0.00	0.00
OC+PG	8.56	0.88	7.18	0.41	0.00	0.00	0.00	0.00	0.00	0.00
OG+PC	2.91	0.88	0.62	1.33	0.00	0.00	0.00	0.00	0.00	0.00
OG+PG	1.77	0.85	0.42	0.39	0.00	0.00	0.00	0.00	0.00	0.00

Table S2 . Execution times for the benchmark cuboid test in minutes (min) for the *ToR-ORd* cellular model in the 8 considered scenarios. A+OC+PC: Adaptive, ODEs on CPU, PDE on CPU; A+OG+PC: Adaptive, ODEs on GPU, PDE on CPU; A+OC+PG: Adaptive, ODEs on CPU, PDE on GPU; A+OG+PG: Adaptive, ODEs on GPU, PDE on GPU; OC+PC: Non-adaptive, ODEs on CPU, PDE on CPU; OC+PC: Non-adaptive, ODEs on CPU, PDE on CPU; OC+PG: Non-adaptive, ODEs on CPU, PDE on GPU; OG+PC: Non-adaptive, ODEs on GPU, PDE on CPU; OG+PG: Non-adaptive, ODEs on GPU, PDE on GPU.

Scenario	Total	Write	ODE	PDE	Reassemble matrix	Refine grid	Derefine grid	Order grid	Update cells	Update state vector
A+OC+PC	11.18	0.46	7.36	2.27	0.10	0.44	0.38	0.10	0.03	0.01
A+OG+PC	6.10	0.46	0.83	2.27	0.10	0.44	0.39	0.10	0.03	1.31
A+OC+PG	9.95	0.49	7.35	0.68	0.10	0.44	0.36	0.09	0.02	0.01
A+OG+PG	4.66	0.47	0.63	0.67	0.11	0.43	0.37	0.09	0.02	1.31
OC+PC	25.11	0.95	22.44	1.68	0.00	0.00	0.00	0.00	0.00	0.00
OC+PG	24.14	0.94	22.53	0.59	0.00	0.00	0.00	0.00	0.00	0.00
OG+PC	3.80	0.91	1.17	1.62	0.00	0.00	0.00	0.00	0.00	0.00
OG+PG	2.29	0.90	0.80	0.51	0.00	0.00	0.00	0.00	0.00	0.00

model dense and sparse regions of subendocardial Purkinje coupling (Fig. 2C). Control volumes inside these regions are treated as isotropic (conductivity values calibrated based on the conduction velocities following[5], as detailed in Supplementary Material section A.4), with a size for both layers equivalent to mesh discretisation element. Both regions are also used to generate a different number of PMJs on the endocardium for the Purkinje network generation method[6]. Finally, the scaling factor map for the I_{Ks} current used in the myocardial cellular *ToR-ORd* model for T-wave personalisation[3] is shown in Figure 2D.

In terms of cellular electrophysiology, the above-mentioned *ToR-ORd* model was used for describing the myocardium, with minor modifications aiming to improve T-wave personalisation[4]: 50% scaling of I_{Kr} conductance, 5-fold scaling of I_{Ks} conductance[7], and reduction of the time constant of L-type calcium channel activation (τ_{jca}) from 75 to 60 ms. For the Purkinje domain, the *Trovato* human Purkinje model[8] was considered. Both ODE systems were solved with a Rush-Larsen scheme and fixed timestep of $\Delta t = 0.01$ ms. The same discretisation step was used to solve the associated PDEs, for a total simulation time of 600 ms. The stimulus protocol was a single pulse applied at the His bundle ($N_{cells} = 25$) of amplitude 40 pA/pF and 2 ms duration. Other monodomain parameters were set to $\beta = 1400$ cm^{-1} and $C_m = 1$ $\mu F/cm^2$.

Next, to generate a full branched Purkinje network that sustains the morphology of the clinical ECG we used the open source Purkinje generation method as described in the work from Berg et al.[6]. The procedure consists of first generating additional PMJs within the dense and sparse regions of the endocardium, where these regions are highlighted in Figure 2C. Next, the new PMJs are connected by the extra branching procedure described in[6] using the minimum Purkinje network as the initial root.

The monodomain conductivities are calibrated to reproduce the conduction velocity (CV) and activation times given by the reduce-order model into a biophysically-detailed one (monodomain) using cable simulations with MONOALG3D following the same CV tuning procedure as described in the work from Costa et al.[5].

Regarding the monodomain parameters utilized for this experiment, the surface-to-volume ratio was set to $\beta = 1400 cm^{-1}$

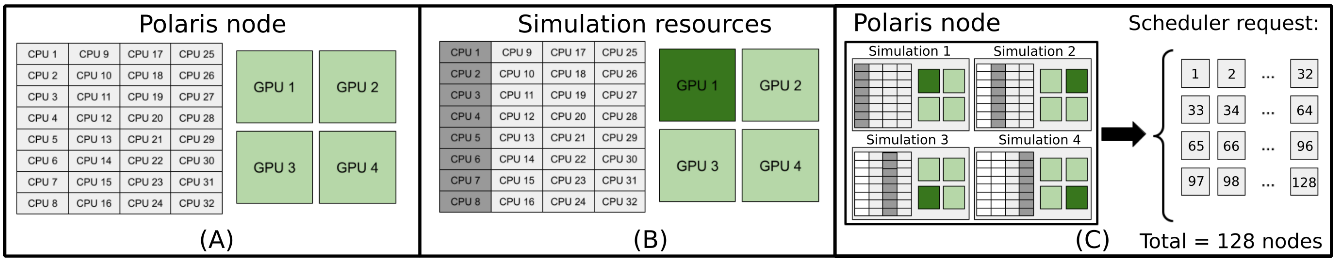

Figure S4 . Strategy to dispatch the patient-specific jobs on Polaris. For each Polaris node a single patient-specific simulation uses 8 CPU cores and 1 GPU. To enable the full usage of the machine resources we allocate 4 concurrent simulations using the MPI batch feature on a Polaris node, where each simulation uses a different GPU. (A) Polaris node CPU/GPU hardware overview. (B) Amount of resources used for one patient-specific simulation on Polaris. (C) Job allocation request to the machine scheduler to run 512 simulations using 128 nodes with the MPI feature.

and the membrane capacitance to $Cm = 1\mu F/cm^2$. The maximum simulation time is $t_{max} = 600ms$ and a time discretisation equal to $dt = 0.01ms$ was used to solve the associated PDEs. For the ODEs system related to the Purkinje and myocardium cellular models, a Rush-Larsen scheme with a fixed timestep of $dt = 0.01ms$ was applied.

To model the cellular dynamics of the myocardium domain, the adjusted version of the *ToR-ORd* human ventricular model[9] was used as previously described, while for the Purkinje domain, the *Trovato* human Purkinje model[8] was consider. The rationale to use both cellular models is that they can be considered the state-of-the-art in terms of human cellular model currently available in the literature.

A.4 Conductivities and conduction velocities

Conductivities were calibrated using monodomain cable simulations to match conduction velocities (CVs), as summarised in Table S3. Of note, all CVs are close to physiological values given by Durrer et al.[10]. The same CV tuning procedure presented in the work from Costa et al.[5] was applied with MonoAlg3D using the *tuneCV* script (https://github.com/rsachetto/MonoAlg3D_C/tree/master/scripts/tuneCV).

Table S3 . Monodomain conductivity values (mS/cm) and conduction velocities (m/s) for the Purkinje and myocardium domains.

	Purkinje		Endocardium layers				Fibre		Sheet		Normal	
	CV_p	σ_p	CV_{dense}	σ_{dense}	CV_{sparse}	σ_{sparse}	CV_f	σ_f	CV_s	σ_t	CV_n	σ_n
Coarse mesh	3	115	1.3	8.81	0.97	5.35	0.65	2.83	0.27	0.92	0.48	1.83
Fine mesh	3	60	1.3	7.78	0.97	4.51	0.65	2.21	0.27	0.54	0.48	1.31

A.5 Patient-specific simulation dispatch strategy

To dispatch the patient-specific simulations on Polaris, we considered that optimal performance for simulations using entirely the GPU was attained with 8 OpenMP threads and 1 GPU device (scenario OG+PG), as informed by the results in Table 1 from the main manuscript.

According to the hardware specification, a Polaris compute node has 32 physical CPU cores and 4 GPU devices. Within this context, we can execute 4 concurrent simulations on each node using 8 CPU cores and 1 GPU each without wasting resources. To enable the 512 simulations, we made a job request for 128 compute nodes, where 4 jobs are concurrently executed on each node using the MPI batch feature (see Figure S4).

A.6 Additional patient-specific results

The results for the 512 biventricular simulation study using the *coarse* mesh setup are presented in Figure S5. These were used to select a pair of Purkinje coupling parameters ($R_{PMJ} = 1029\ k\Omega, N_{PMJ} = 43$) providing both a physiological anterograde PMJ delay (Fig.S5A) and a sufficiently accurate approximation of the ECG (Fig.S5B). Figures S5D and S5F highlight the activation of a PMJ site exhibiting an anterograde delay of 5.61 ms and a similar activation pattern as the one presented in Figure 5F from the main manuscript. Furthermore, on average, the anterograde PMJ delay was around 4.10 ± 2.34 ms, in agreement with the physiological range reported in the literature that is around $4-14$ ms[11]. Regarding the ECG approximation, we achieved an average PCC of 0.78 for this combination of parameters.

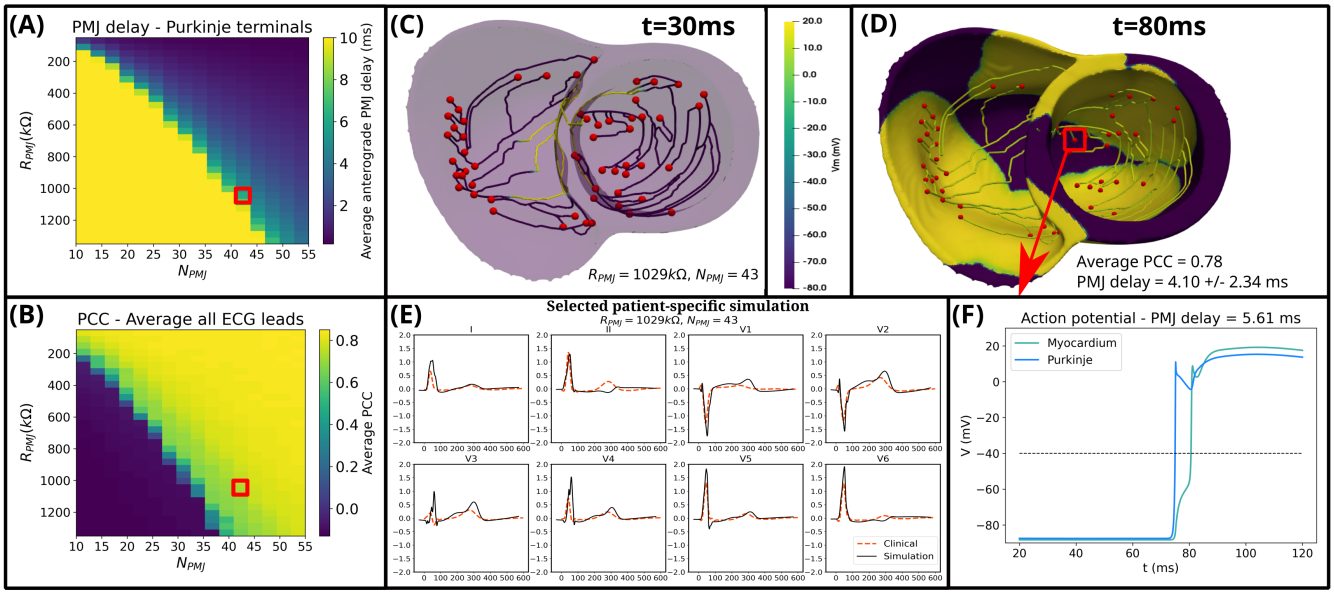

Figure S5 . Results for the 512 patient-specific simulations with the *coarse* mesh. (A) Average anterograde PMJ delay across all Purkinje terminals. (B) Average PCC across all leads between the clinical and simulated ECG. Selected Purkinje coupling parameters $(R_{PMJ} = 1029\ k\Omega, N_{PMJ} = 43)$ are highlighted by red squares. (C-D) Selected patient-specific simulation with an average PCC of 0.78 in ECG reconstruction and average anterograde PMJ delay of $4.10 \pm 2.34\ ms$, at times $t = 30$ and $t = 80\ ms$, respectively. (E) Comparison between clinical and simulated ECGs. (F) Action potential upstrokes for the PMJ site highlighted in panel (D) with anterograde PMJ delay of 5.61 ms, at the terminal Purkinje volume (blue) and its closest coupled myocardial volume (lime). The 3D models in panels (C) and (D) were generated using the last version of MONOALG3D (https://github.com/rsachetto/MonoAlg3D_C) and visualised with the Paraview tool version 5.13.2.

A.7 Convergence analysis for the cuboid benchmark

A convergence analysis based on the benchmark proposed by Niederer et al.[12] was done for the cuboid benchmark simulation using the same settings as described in section *Computational simulations - Benchmark myocardium cuboid* from the main manuscript using MONOALG3D and OPENCARP[13], another open-source cardiac electrophysiology solver.

In Figure S6A, the local activation time across the highlighted red line is computed for different space discretisation values (500 μm, 250 μm, 100 μm and 50 μm) and represented in Figure S6B. Based on these results, we can verify the two solvers robustness as the local activation time converges as we increase the mesh refinement as expected.

Furthermore, as shown in Figure S6B, the solution with a spatial discretisation of $h = 500\ \mu m$ exhibits a significant discrepancy in the local activation time. Specifically, when comparing the local activation time at the last element of the reference red line in Figure S6A to the same solution with $h = 50\ \mu m$ on both solver. Relative errors of approximately 219.28 % and 220.07 % are observed for MONOALG3D and OPENCARP, respectively. In contrast, this error decreases for finer discretisations, dropping to 44.12 % and 41.58 % with $h = 250\ \mu m$ and to 5.56 % and 2.04 % with $h = 100\ \mu m$, respectively.

These findings indicate that a mesh resolution of 500 μm provides a high relative error on both solvers and should be avoided, especially under pathological scenarios where accuracy is critical. In these scenarios, finer discretisations (e.g., 250 μm or better) are more appropriate.

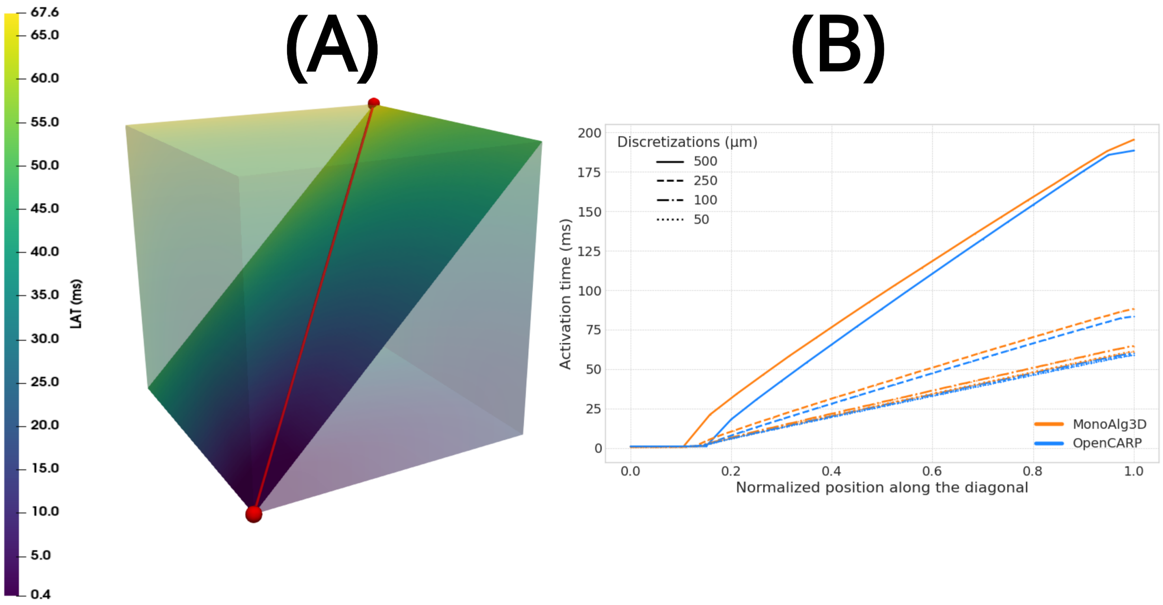

Figure S6 . (A) Local activation time map of the cuboid benchmark simulation solved with MONOALG3D and using $\Delta t = 0.02$ ms and $h = 62.5$ μm with the highlighted red line used to compute the activation times presented in panel (B). (B) Local activation time across the red line presented in panel (A) considering two cardiac eletrophysiology solvers, MONOALG3D (orange) and OPENCARP (blue) when different mesh resolutions ranging from 50 μm to 500 μm are used. The red line distance was normalised and the 3D model in panel (A) was generated using the last version of MONOALG3D and visualised with the Paraview tool.

References

1. Banerjee, A. *et al.* A completely automated pipeline for 3D reconstruction of human heart from 2D cine magnetic resonance slices. *Philos. Transactions Royal Soc. A* **379**, 20200257 (2021).
2. Camps, J. *et al.* Inference of ventricular activation properties from non-invasive electrocardiography. *Med. Image Analysis* **73**, 102143 (2021).
3. Camps, J. *et al.* Digital Twinning of the Human Ventricular Activation Sequence to Clinical 12-lead ECGs and Magnetic Resonance Imaging Using Realistic Purkinje Networks for in Silico Clinical Trials. *Med. Image Analysis* **94**, 103108 (2024).
4. Camps, J. *et al.* Harnessing 12-lead ECG and MRI data to personalise repolarisation profiles in cardiac digital twin models for enhanced virtual drug testing. *Med. Image Analysis* **100**, 103361 (2025).
5. Costa, C. M., Hoetzl, E., Rocha, B. M., Prassl, A. J. & Plank, G. Automatic parameterization strategy for cardiac electrophysiology simulations. In *Computing in Cardiology 2013*, 373–376 (IEEE, 2013).
6. Berg, L. A. *et al.* Enhanced optimization-based method for the generation of patient-specific models of Purkinje networks. *Sci. Reports* **13**, 11788 (2023).
7. Doste, R., Coppini, R. & Bueno-Orovio, A. Remodelling of potassium currents underlies arrhythmic action potential prolongation under beta-adrenergic stimulation in hypertrophic cardiomyopathy. *J. Mol. Cell. Cardiol.* **172**, 120–131 (2022).
8. Trovato, C. *et al.* Human Purkinje in silico model enables mechanistic investigations into automaticity and pro-arrhythmic abnormalities. *J. Mol. Cell. Cardiol.* **142**, 24–38 (2020).
9. Tomek, J. *et al.* Development, calibration, and validation of a novel human ventricular myocyte model in health, disease, and drug block. *Elife* **8**, e48890 (2019).
10. Durrer, D. *et al.* Total excitation of the isolated human heart. *Circulation* **41**, 899–912 (1970).
11. Behradfar, E., Nygren, A. & Vigmond, E. J. The role of Purkinje-myocardial coupling during ventricular arrhythmia: a modeling study. *PLoS One* **9**, e88000 (2014).

12. Niederer, S. A. *et al.* Verification of cardiac tissue electrophysiology simulators using an N-version benchmark. *Philos. Transactions Royal Soc. A* **369**, 4331–4351 (2011).

13. Plank, G. *et al.* The openCARP simulation environment for cardiac electrophysiology. *Comput. Methods Programs Biomed.* **208**, 106223 (2021).
